# Epigenetic modulators provide a path to understanding disease and therapeutic opportunity

**DOI:** 10.1101/gad.351444.123

**Published:** 2024-06-01

**Authors:** Madison A. Honer, Benjamin I. Ferman, Zach H. Gray, Elena A. Bondarenko, Johnathan R. Whetstine

**Affiliations:** 1 Cancer Epigenetics Institute, Fox Chase Cancer Center, Philadelphia, Pennsylvania 19111, USA;; 2 Nuclear Dynamics and Cancer Program, Fox Chase Cancer Center, Philadelphia, Pennsylvania 19111, USA;; 3 Institute for Cancer Research, Fox Chase Cancer Center, Philadelphia, Pennsylvania 19111, USA;; 4 Biomedical Sciences Program, Lewis Katz School of Medicine, Temple University, Philadelphia, Pennsylvania 19140, USA

**Keywords:** bromodomain, DNA and histone modifications, DNA methylation, SWI/SNF, acetylation, chromatin remodeling, demethylase, ecDNA, epigenetics, transcription

## Abstract

The discovery of epigenetic modulators (writers, erasers, readers, and remodelers) has shed light on previously underappreciated biological mechanisms that promote diseases. With these insights, novel biomarkers and innovative combination therapies can be used to address challenging and difficult to treat disease states. This review highlights key mechanisms that epigenetic writers, erasers, readers, and remodelers control, as well as their connection with disease states and recent advances in associated epigenetic therapies.

## Abstract (precis)

In this review, Honer et al. unravel our mechanistic understanding of how DNA and chromatin modifiers, like writers, erasers, readers, and remodelers, function and contribute to human disease. They further highlight recent advances in both monotherapies and combination therapeutic approaches that target these epigenetic modulators to combat various diseases.

The field of epigenetics addresses heritable phenotypes generated through nongenetic perturbation to the genome and the mechanistic processes occurring above the nucleotide sequence of DNA. For example, direct modifications to DNA, the organization of DNA around histone octamers making up chromatin, and the direct ability of post-translational modifications (PTMs) to form on histone tails impact heritable traits such as gene regulation and cell fate decisions. Along with being heritable, epigenetic states can be dynamic and reversible (Jenuwein and Allis 2001). Emerging findings and the use of cutting-edge technologies have provided insights into the physiological processes that epigenetic modulators control. Numerous studies have documented aberrant expression as well as germline and somatic mutations in protein-coding genes influencing the PTMs, regulation of the organizational landscape of the genome, and in turn pathogenesis, which has revealed biomarkers and novel therapeutic targets in order to combat a host of diseases (Dawson and Kouzarides 2012; Rando and Chang 2012; Sen et al. 2016; Dobson et al. 2018; Cavalli and Heard 2019; Jaiswal and Ebert 2019; Alonso-Curbelo et al. 2021; Wu et al. 2024). This review briefly discusses the history and discovery of human epigenetic modulators and highlights their relevance to human disease, but it must be noted that these discoveries are often built on fundamental research from using model organisms to human cell models. Here, we highlight each major class of modulators—writers, erasers, readers, and remodelers—and specific examples within these classes regarding their regulation, function, cross-talk dynamics, and contribution to disease, as well as prospects for future disease diagnosis and therapeutic intervention. With the immense amount of data and progress in the field, we unfortunately are not able to cite all that have shaped the general understanding but do highlight key studies related to the points being covered.

## Types of epigenetic modulators

**Figure 1.**
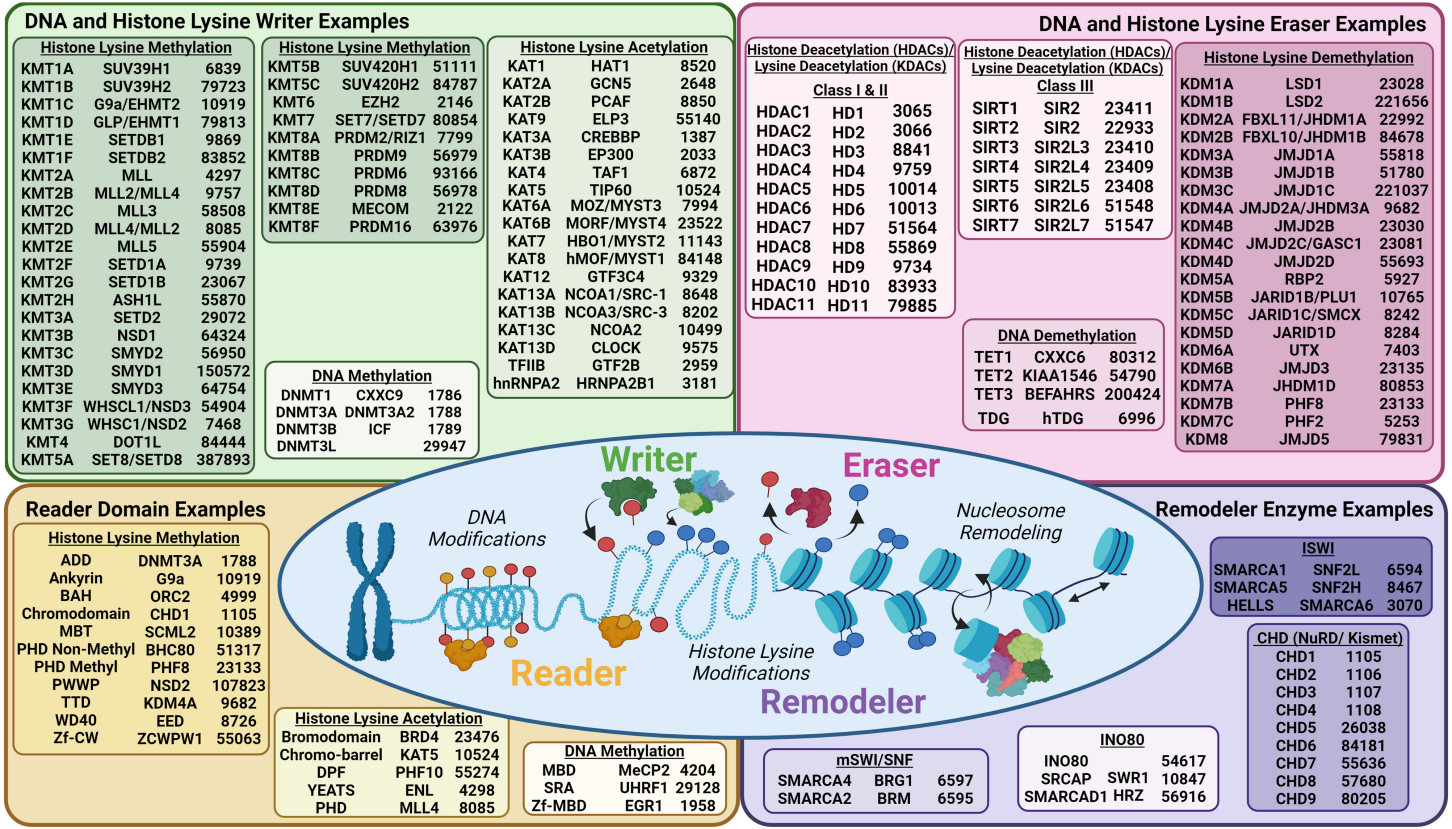
DNA and histone lysine writers, erasers, reader domains, and remodelers. The schematic notes either official symbol/aliases or recognized nomenclature with the appropriate gene ID for each writer, eraser, and remodeler example. Example reader domain types are noted with their reader domain type, gene name, and associated gene ID. (BAH) Bromo-adjacent homology, (MBT) malignant brain tumor, (PHD) plant homeodomain, (TTD) tandem Tudor domain, (Zf-CW) zinc finger CW, (DPF) double-PHD finger, (MBD) methyl-CpG binding domain, (SRA) SET- and RING-associated, (Zf-MBD) zinc finger methyl-CpG binding domain

The epigenetic modulators that alter DNA and histones are grouped by their enzymatic or biochemical function, which includes writers, erasers, readers, and remodelers (Fig. 1).

### Writers

Writers are enzymes that actively place chemical modifications on DNA, histones, and nonhistone proteins (Fig. 1). The DNA modification 5-methylcytosine (5mC) was discovered in 1948 (Hotchkiss 1948), and later the asymmetric presence of the CpG methylation was linked to epigenetic regulation (Holliday and Pugh 1975; Razin and Riggs 1980). In the mid-1970s, the first 5mC enzyme was purified from HeLa cells and at that time was called DNA methylase but is now known as DNMT1 (Roy and Weissbach 1975). This discovery resulted in additional DNA methyltransferases (DNMTs) being discovered (Okano et al. 1998; Aapola et al. 2000). These writer enzymes catalyze the transfer of a methyl group from S-adenosyl-L-methionine (SAM) to cytosine in DNA to form 5mC (Lyko 2018). For histones, acetylation levels were linked to changes in transcriptional activity that were documented by Allfrey and Mirsky (1964). However, it was not until 1996 that the first histone lysine acetyltransferase (HAT or KAT), p55, was discovered by Allis and colleagues (Brownell et al. 1996) in *Tetrahymena thermophila.* In the same time line, the Sternglanz and Gottschiling groups (Kleff et al. 1995; Parthun et al. 1996; Marmorstein and Zhou 2014) discovered HAT1, which acetylates cytoplasmic histones in humans. KATs function by transferring the acetyl group from acetyl coenzyme A (acetyl co-A) to the ε-amino group of conserved lysine residues on histones to form ε-N-acetyl lysine (Lee et al. 2007; Marmorstein and Zhou 2014). Shortly after KATs were resolved, the first histone lysine methyltransferase (KMTs), KMT1A/SUV39H1, was discovered in 2000 by Jenuwein and colleagues (Rea et al. 2000). This seminal discovery paved the way for the discovery of numerous other KMTs, which catalyze the transfer of a methyl group from SAM to a lysine residue, allowing for monomethylation (me1), dimethylation (me2), or trimethylation (me3) (Black et al. 2012; Hyun et al. 2017; Husmann and Gozani 2019; Jambhekar et al. 2019). The degree of lysine methylation and genomic location have distinct consequences, and the balance between KATs and KMTs can have opposing or similar mechanisms influencing DNA-dependent processes such as regulating replication, gene expression, cell cycle, protein–DNA interactions, and nuclear dynamics. This interplay also applies to other writers of histone modifications (Tahiliani et al. 2009; Bannister and Kouzarides 2011). Here, we focus on writers of DNA methylation and histone lysine acetylation and methylation.

### Erasers

**Figure 2.**
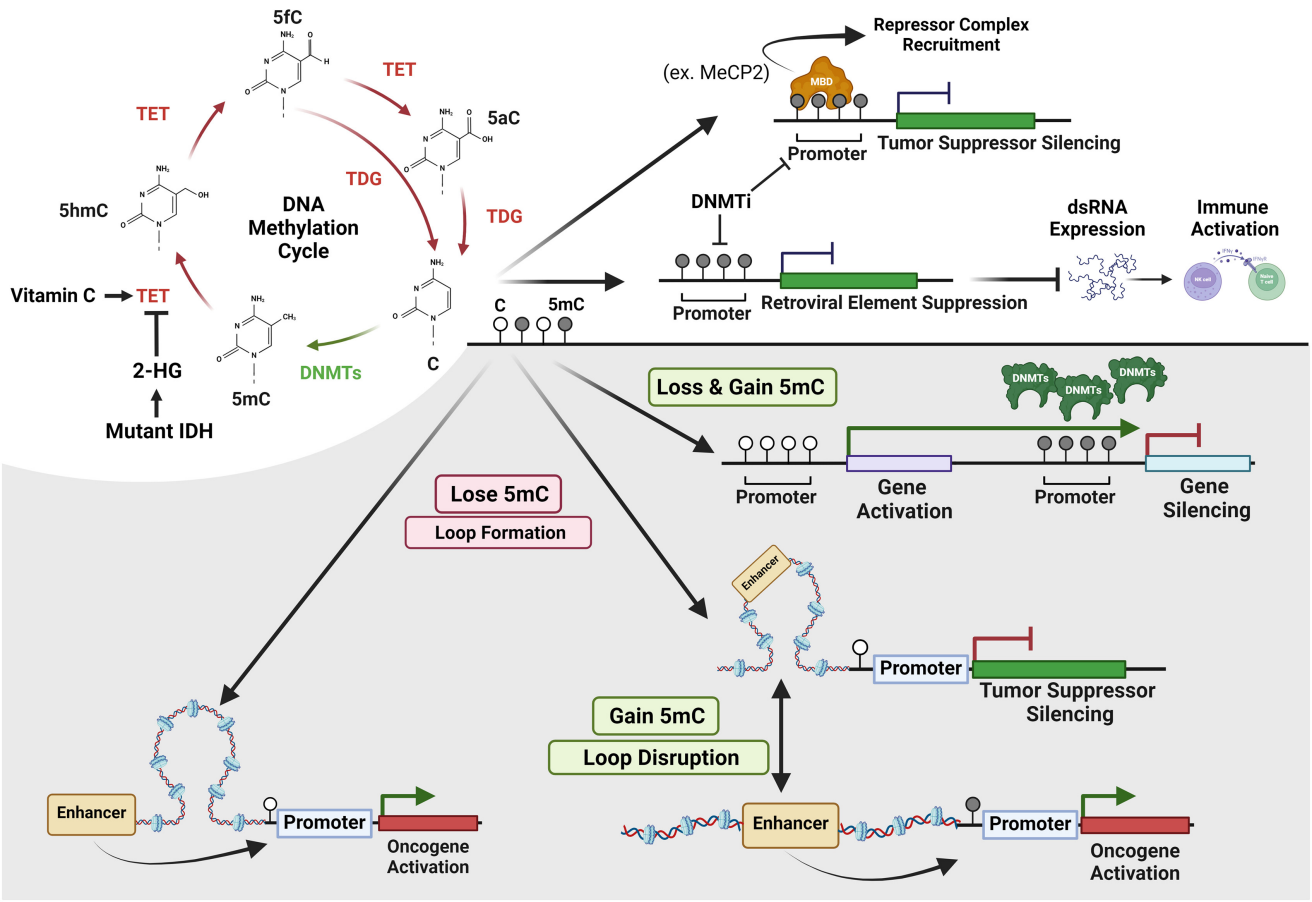
DNA methylation regulation. The DNA methylation cycle and cofactors that alter methylation are noted. The examples illustrate how DNA methylation controls tumor suppressors and retrotransposable elements; how 5mC impacts loops, gene activation, and repression; and how hypomethylation and hypermethylation coexist at a locus.

Eraser enzymes actively remove their targeted modification (Fig. 1). In 1996, the first histone deacetylase (HDAC), HDAC1 (also known as lysine deacetylase [KDAC]), which has a yeast ortholog reduced potassium dependency 3 (Rpd3), was purified by Schreiber and colleagues (Taunton et al. 1996) from cow protein extracts. This discovery resulted in a collection of HDACs subsequently being identified. HDACs catalyze the removal of the ε-amino acetyl group from lysine residues (Seto and Yoshida 2014; Ho et al. 2020). HDACs can be divided into two families based on their conserved deacetylase domain and cofactor dependencies. The zinc-dependent deacetylases consist of three different subclasses (classes I, II, and IV) (Park and Kim 2020). Class II HDACs are further divided into class IIa and class IIb based on their domain compositions. Class III HDACs (Sir2 regulator family) function through an NAD^+^-dependent mechanism and contain seven proteins (Seto and Yoshida 2014). After HDACs were discovered, there was a delay in discovering the lysine demethylases (KDMs) (Black et al. 2012). In 2004, Shi et al. (2004) uncovered the first KDM, LSD1, belonging to the flavin adenine dinucleotide (FAD)-dependent amine oxidase family. This discovery spurred a series of studies that resolved an additional demethylase family called the Jumonji C (JmjC) enzymes (Tsukada et al. 2006; Black et al. 2012). JmjC family members are able to remove all methylation labels and require three key cofactors: oxygen, iron(II) [Fe(II)], and α-ketoglutarate (α-KG) (Black et al. 2012). These discoveries were followed by the resolution of the enzymes that hydroxylate 5-methyl-C (5mC to 5hmC) and then demethylate DNA (Tahiliani et al. 2009; Wu and Zhang 2017). Active DNA demethylation of the paternal genome at fertilization was discovered by Walter and Haaf in 2000 (Mayer et al. 2000; Oswald et al. 2000). However, demethylation remained controversial until 2009–2011, when the ten-eleven translocation–thymine DNA glycosylase (TET–TDG) pathway was discovered. In active demethylation (replication-independent), TET dioxygenases sequentially oxidize 5mC to 5-hydroxymethylcytosine (5hmC), then 5-formylcytosine (5fC), and then 5-carboxylcytosine (5caC) (Tahiliani et al. 2009). 5fC and 5caC are then removed by thymine DNA glycosylase (TDG) through base excision repair (BER) (He et al. 2011; Maiti and Drohat 2011; Bellacosa and Drohat 2015), while Nei-like 1 DNA glycosylase (NEIL1) can also remove 5caC and stimulate TDG activity (Fig. 2; Slyvka et al. 2017). In passive DNA demethylation (replication-dependent), TET proteins convert 5mC to 5hmC, which is poorly recognized by the DNMT1/UHRF1 complex that methylates DNA (Kriaucionis and Heintz 2009; Tahiliani et al. 2009; Ito et al. 2010). Passive demethylation is linked to replication and may affect gene expression by regulating imprinting (Hackett et al. 2013).

### Readers

As modifications are laid down by epigenetic writers, they are recognized by epigenetic reader proteins with modification-specific domains (Fig. 1; Strahl and Allis 2000; Musselman et al. 2012). The first reader structure to be resolved was the bromodomain in lysine acetyltransferase (KAT) P/CAF (EP300/CBP-associated factor) (Dhalluin et al. 1999). Histone acetylation readers include bromodomains (BRDs), double-PHD finger domains (Zeng et al. 2010; Qiu et al. 2012; Sabari et al. 2017), and Yeats domains discovered in yeast (Tamkun et al. 1992; Le Masson et al. 2003; Dreveny et al. 2014), while methyl lysine readers consist of numerous domain types (Fig. 1; Yun et al. 2011). In contrast, there are three families of readers that bind methylated DNA, including the methyl CpG binding domain (MBD) family that works to silence transcription (Du et al. 2015), SET- and RING-associated (SRA) domain proteins, and zinc finger (ZnF) proteins (Fig. 1; Moore et al. 2013). Collectively, these proteins and their associated domains are critical for recognizing epigenetic marks for regulation of cellular processes.

### Remodelers

**Figure 3.**
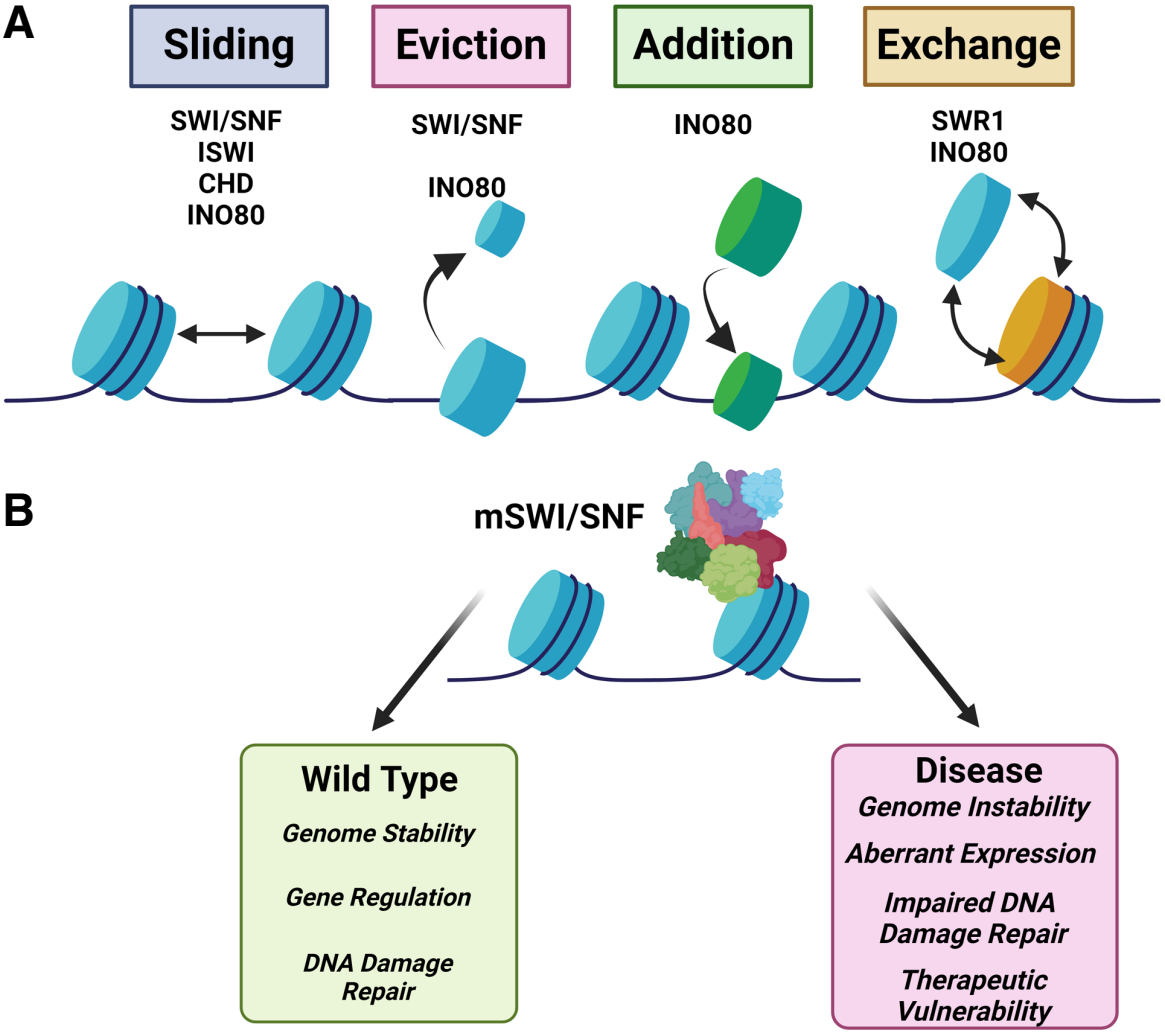
Remodeler activities and the impact of mSWI/SNF mutations. (*A*) Illustration highlighting the various remodeler families and associated activities. (*B*) Schematic showing the impact of wild-type mSWI/SNF and the impact of mutations or misregulation.

Chromatin remodelers are multisubunit complexes that are dependent on energy from ATP hydrolysis to reposition, slide, eject, or alter the composition of nucleosomes (Fig. 3). They directly modify chromatin accessibility and nucleosome positioning in order to regulate transcription, DNA repair, and replication (Fig. 3). To date, four unique families of chromatin remodelers have been identified: switch/sucrose nonfermentable (SWI/SNF), INO80, ISWI, and CHD (Valencia and Kadoch 2019; Reyes et al. 2021). The SWI/SNF family promotes chromatin accessibility through establishing nucleosome-depleted regions and positioning for DNA repair, recombination, and transcription factor binding (Clapier et al. 2017). SWI/SNF complexes were first characterized in yeast (Carlson et al. 1984; Neigeborn and Carlson 1984; Stern et al. 1984; Côté et al. 1994) and later characterized in *Drosophila* (Tamkun et al. 1992) and mammals (Imbalzano et al. 1994; Kwon et al. 1994). Later, it was shown that certain remodeler complexes can specifically swap out H2A with histone variant H2AZ in nucleosome arrays (Mizuguchi et al. 2004; Clapier et al. 2017). This exchange of histone variants is accomplished by the INO80 family, consisting of INO80 and SWR1. INO80 promotes DNA translocation and H2A–H2B dimer exchange (Brahma et al. 2017), induces unwrapping of DNA on nucleosomes near the entry site, and disrupts histone interactions that lead to the partial exposure of H2AZ–H2B dimers (Eustermann et al. 2018). These enzymes also contain other critical components, including reader domains, which guide their targeting. The subsequent sections highlight specific examples for each subclass of epigenetic modulators in regard to their mechanistic function and link(s) to disease.

## Epigenetic modulators and their mechanistic link to disease

**Figure 4.**
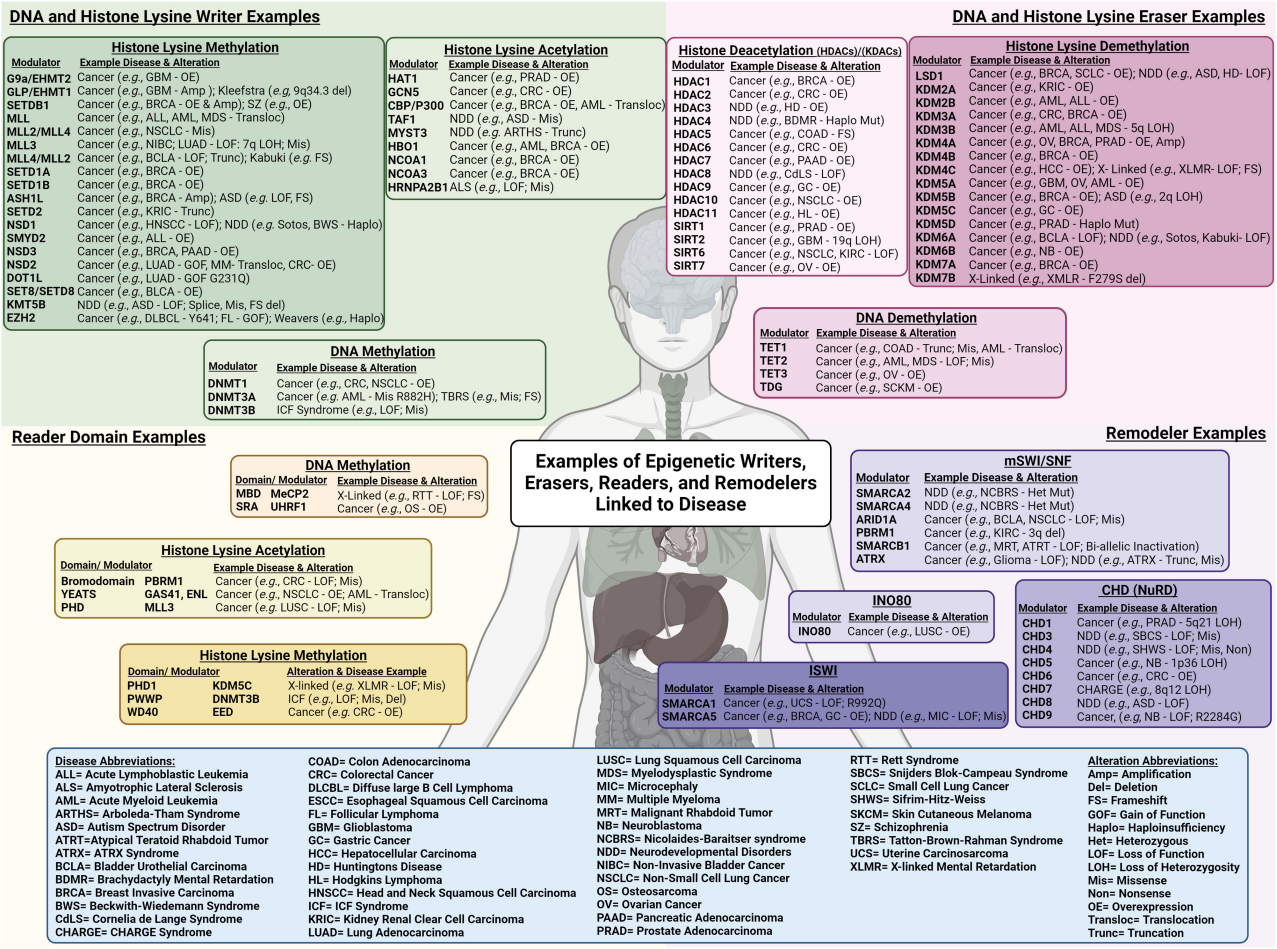
DNA and histone lysine writers, erasers, reader domains, and remodelers linked to disease. Epigenetic modifying enzymes are linked to cancer, disease, and genetic disorders. Genetic alterations (mutations, translocations, etc.) and/or misexpression of epigenetic modulators are prevalent across numerous disease states and are mechanistically linked to the associated disease. Examples for each modulator class are shown with the example alteration and associated disease or disorder. All abbreviations are noted in the blue box.

Developmental, aging, and immunological disorders as well as cancer are some of the most studied diseases associated with epigenetic misregulation (Cavalli and Heard 2019). A wide range of epigenetic modulators are linked to an array of diseases (Figs. 1, 4). Emerging findings reveal insights into the disease mechanism(s) and provide novel therapeutic targets to combat diseases. Studies have revealed an association between genetic alterations (e.g., point mutations, loss of heterozygosity, single-nucleotide polymorphisms, amplifications, and rearrangements) and numerous disease states. Genome-wide association studies have also linked epigenetic modulators’ expression and genetic variants to altered gene expression associated with disease (Lawrence et al. 2013; Van Rechem and Whetstine 2014; Cavalli and Heard 2019; Alexandrov et al. 2020). Genetics typically provide the strongest degree of evidence for disease association. However, direct testing of the associations is required, which is an active area of investigation and needs further development in the field. In this review, we highlight how alterations (genetic and regulatory) in each epigenetic modulator class impact function and disease. The selected examples in this review aim to highlight their function, the impact on epigenetic modulator cross-talk, and their molecular contribution to normal and disease states. Although this review is unable to discuss the full array of epigenetic modulator mutations or misregulation observed across diseases, we created a figure that highlights a number of examples that illustrate the breadth of alterations associated with diseases (Fig. 4). Most of these examples are discussed in the remainder of this review. We also recommend the following reviews for more in-depth information for each class and their disease association: For writers, see Rasmussen and Helin (2016) for DNA and Husmann and Gozani (2019) and Nitsch et al. (2021) for histones; for erasers, see Lyko (2018) for DNA and Dimitrova et al. (2015) and Bondarev et al. (2021) for histones; for remodelers, see Valencia et al. (2023) and Gourisankar et al. (2024); and for readers, see Mahmood and Rabbani (2019) and Cipriano et al. (2020).

## DNA methylation

### DNA methylation writers

There are three “writers” of DNA methylation (DNMT1, DNMT3A, and DNMT3B) (Fig. 1) that use S-adenosyl-methionine for the methyl donation to cytosine (5mC) (Lyko 2018). DNMT3A and DNMT3B are de novo DNA methyltransferases, whereas DNMT1 can facilitate DNA methylation either de novo or for methylation maintenance. DNMT3L is a DNMT3-like protein that lacks a catalytic domain and associates with DNMT3A and DNMT3B, which in turn helps their catalytic activities (Chédin et al. 2002; Suetake et al. 2004; Nishiyama and Nakanishi 2021). In contrast, DNMT2 is an RNA methyltransferase. Dysregulation of DNMTs and altered DNA methylation patterns impact disease mechanisms and are used as clinical biomarkers. Disease-associated alterations are often characterized by genome-wide hypomethylation leading to gene activation, which is accompanied by focal hypermethylation of CpG island promoters to repress gene activity (Fig. 2; Baylin and Jones 2016). Many cancers harbor mutations in genes that encode for components of the epigenetic machinery and are central to establishing normal chromatin and DNA methylation patterns (Hanahan 2022). For example, mutation or overexpression of *DNMT1* drives colorectal cancer and non-small-cell lung cancer (NSCLC) (Zhang et al. 2020), whereas *DNMT3A* and *TET2* mutations or *TET1* translocations are linked with acute myeloid leukemia (AML) and myelodysplastic syndrome (MDS) (Fig. 4; De Carvalho et al. 2010; Wu and Zhang 2011; Rasmussen and Helin 2016). While the associated diseases display a multitude of differentially expressed genes and DNA methylation patterns, selected examples are highlighted below to illustrate how DNA methylation writers impact gene expression and disease.

Groups of CG repeats, called CpG islands, are frequently hypermethylated on gene promoters in cancer and silence tumor suppressor genes, including the *retinoblastoma* gene (*RB1*) (Fig. 2; Greger et al. 1989; Gazzoli et al. 2002; Jones and Baylin 2007). Hypermethylation of tumor suppressors and immune response genes and the fact that DNMTs are overexpressed in cancer have made them therapeutic targets (e.g., DNMT1) (Fig. 2; Mizuno et al. 2001; Esteller 2008; Zhang et al. 2020; Pappalardi et al. 2021). While CpGs can be hypermethylated in tumors, cancer cells also have global DNA hypomethylation across megabases (called partially methylated domains [PMDs]) linked to genomic instability and activation of proto-oncogenes (Nishiyama and Nakanishi 2021). Mutations in *DNMTs* contribute to these alterations (Zhang et al. 2020). For example, *DNMT3A* mutations frequently occur in myeloid malignancies (∼20% of adult hematopoietic malignancies; R882 mutations) (Fig. 4; Ley et al. 2010). These malignancies are characterized by DNA hypomethylation, increased recruitment of active histone modulators at enhancer elements, aberrant activation of leukemic stemness genes (e.g., *Hoxa* gene cluster and HOX cofactors) (Lu et al. 2016), and recruitment of polycomb group (PcG) proteins to genes associated with differentiation of HSCs (Fig. 2; Koya et al. 2016; Jeong et al. 2018; Zhang et al. 2020; Nishiyama and Nakanishi 2021). These data demonstrate the importance of each DNMT member and how they could have different impacts depending on the genomic location and cancer type.

Although hypomethylation and hypermethylation being in the same tumor seems contradictory, studies have shed light onto why and how they can occur within the same genome. These studies identified that hypomethylation of a gene locus leads to activation of longer transcripts that overlap downstream gene promoters that are enriched for CpG islands (Fain et al. 2021). Transcriptional elongation into the downstream genes coincides with increased DNA methylation placed by the enzyme responsible for methylation maintenance, which promotes their hypermethylation and silencing (Fig. 2; Fain et al. 2021). Therefore, displaced DNA methylation can provide instruction for the DNA methylation writers. Studies delving into the DNMTs’ specific contribution to the regulation of the genome and how their impact reverberates across the epigenetic landscape will be important to properly leverage the collection of therapeutics against DNMTs.

A DNMT inhibitor, 5-azacitidine (vidaza), obtained FDA approval for the treatment of MDS in 2004 (Kaminskas et al. 2005). In turn, numerous other trials and approvals for it and other DNMT inhibitors in additional hematologic cancers occurred (Zhang et al. 2022). Since then, novel therapeutics and combinations have emerged. In 2020, decitabine and cedazuridine, a cytidine deaminase inhibitor (Inqovi), was approved for MDS treatment (Lopez et al. 2022). A DNMT1-specific inhibitor, GSK3685032, was recently used to induce gene activation and hypomethylation in AML cell lines and was effective in human AML xenograft models (Pappalardi et al. 2021). This inhibitor could be used to treat imbalance in the expression of DNMT1 and TET1, a DNA methyl-eraser. Their imbalance leads to a CpG island methylator phenotype (CIMP) that results in tumor suppressor genes being turned off and is frequently observed in colorectal tumors (Tricarico et al. 2023). Additionally, aberrant DNA methylation can lead to immune evasion (Roulois et al. 2015; Hu et al. 2021). DNA methylation suppresses endogenous retroviral (ERV) elements and IFNγ signaling and dampens immune recognition, which can be reversed with DNMT inhibitors (Fig. 2; Roulois et al. 2015; Hu et al. 2021). 5-aza-2-cytidine (5-AZA-CdR) and decitabine (5-aza-2′-deoxycytidine) enhance human IFNγ^+^ T-cell activation and proliferation, leading to increased activity of cytotoxic T-cells (Zhang et al. 2022). DNA hypomethylation also enhances *PD-L1* expression in tumor cells and increases the expression of immune-related genes and T-cell infiltration (Roulois et al. 2015; Li et al. 2017). For these reasons, DNMTis have been used in combination with monoclonal antibody immune therapies such as anti-CD47 (magrolimab), anti-CD3 (visilizumab), anti-CD123 (talacotuzumab), anti-TIM-3 (sabatolimab), or anti-PD-1 (camrelizumab) that have advanced to phase III and IV clinical trials. While the data suggest that there could be clinical benefits for DNMT inhibitors plus immune checkpoint inhibitors, a recent study demonstrated that guadecitabine plus atezolizumab in metastatic bladder cancer did not guarantee a better patient response, which highlighted the importance for additional biomarkers to predict benefit or even adverse effects (Jang et al. 2023).

Recent evidence demonstrated the potential for using DNMT ihibitors in combination with antibody–drug conjugates (ADCs). For example, decitabine increases the expression of an emerging drug target in prostate cancer, the immune checkpoint antigen CD276/B7 homolog 3 (B7-H3). The combination of decitabine and ADC DS-700a targeting B7-H3 resulted in significantly enhanced response in advanced prostate cancer models (Yamada et al. 2023). Therefore, DNMT inhibition may be a promising therapeutic target to sensitize B7-H3-low prostate cancer to DS-700a treatment through increasing target expression of B7-H3. These examples highlight the therapeutic potential of combining DNMT ihibitors and other epigenetic therapies.

Although understudied, DNMT inhibitors could be useful for other disease treatments. For example, decitabine treatment lowers the promoter methylation of *COX2*, a gene that promotes proliferation in human heart mesenchymal stem cells (HMSCs). Therefore, decitabine may be useful to treat cardiovascular-related diseases where stimulation of cardiac cell proliferation would be beneficial (Sun et al. 2020). Earlier studies also demonstrated the use of decitabine to treat atherosclerosis and coronary heart disease by increasing expression of ERα, ERβ, and COL15A1 in smooth muscle and endothelial cells (Shi et al. 2022). Deciphering a mechanistic understanding of how these drugs work in the heart and other organs will be important in the coming years.

### DNA methylation erasers

Disruption of DNA methylation eraser enzymes also contributes to disease (Figs. 2, 4; Cheng et al. 2019). The TET proteins, responsible for hydroxylating 5mC to generate 5hmC, 5fC, 5caC, and ultimately demethylation (Fig. 2), are associated with leukemia. For example, TET2-specific mutations are observed in 15% of patients with myeloid cancers, including MDS and AML (Delhommeau et al. 2009), and 50% of CML patients (Fig. 4; Kosmider et al. 2009). Most *TET2* mutations are loss of function (LOF), suggesting that defects in active DNA demethylation may promote hematopoietic malignancies (Ko et al. 2010). Supporting this premise, TET2 deficiency in mice results in increased self-renewal of hematopoietic stem or progenitor cells and may lead to malignancy (Li et al. 2011; Moran-Crusio et al. 2011). Reduced expression of TET family proteins results in a significant reduction of 5hmC, 5fC, and 5caC in human breast, liver, lung, pancreatic, and prostate cancers compared with normal tissues (Fig. 4; Yang et al. 2013). TET2 deficiency in diffuse large B-cell lymphoma (DLBCL) leads to hypermethylation in germinal center B-cells and links to transcriptional repression of antigen presentation genes or interferon pathway genes via promoter hypermethylation and loss of enhancer 5hmC (Rosikiewicz et al. 2020). Mutations in *isocitrate dehydrogenase 1* and *2* (*IDH1*/*IDH2*) produce the oncometabolite 2-hydroxyglutarate (2-HG) that inhibits TETs, which in turn promote global hypermethylation (Fig. 2). In *IDH* mutant gliomas, aberrant DNA methylation leads to disruption of an insulator near the *PDGFRA* oncogene, which leads to its activation (Fig. 2; Flavahan et al. 2016). In gliomas and leukemia, *IDH* mutations are mutually exclusive to *TET*-inactivating mutations (Figueroa et al. 2010; Rahme et al. 2023). Consistent with the need to maintain TET function in order to avoid cancer, two clinical trials are leveraging vitamin C, which activates TET2 (Qi et al. 2020), in *TET2* mutant hematologic malignancies (NCT03397173 and NCT03433781) (Fig. 2). In fact, a trial recently demonstrated in a family with a predisposition to lymphoma due to a truncating *TET2* germline mutation that vitamin C diminished the methylation and expression differences when compared with family members without the mutation (Taira et al. 2023). Consequently, metabolite and cofactor presence could impact the DNMT/TET balance. Therefore, the metabolic profile and metabolic state of cells could ultimately shape the effectiveness of therapies and provide a platform to leverage cofactors as well.

*TET2* mutations are also prevalent in clonal hematopoiesis (CH), somatic mutations that arise from clonal hematopoietic stem and progenitor cell (HPSC) expansion and contribute to immune dysregulation (Steensma et al. 2015). *TET2* mutations have been implicated in several of the CH-associated secondary diseases, especially cardiovascular diseases. For example, one study found that *TET* knockout causes DNA hypermethylation of *WNT* inhibitor genes, leading to hyperactivated WNT signaling and defects in cardiac mesoderm patterning. This study demonstrated that inhibition of all three TET enzymes leads to defective cardiac progenitor cell differentiation that formed cardiomyocytes with altered mesodermal patterning (Lan et al. 2021). Using a chronic heart failure model, researchers also showed that hematopoietic- or myeloid-specific *Tet2* depletion impaired cardiac remodeling and function, likely through enhanced inflammasome activity, a signaling cascade contributing to several diseases (Sano et al. 2018). These findings suggest direct mechanisms of TETs as critical regulators of CH-related cardiovascular diseases.

The loss of TET enzymes also impacts genome stability. In mouse oocytes, TET1 loss leads to increased DNA damage, insufficient DNA repair, and genomic instability (Yamaguchi et al. 2012), whereas *TET2* and *TET3* double-knockout cells resolve DNA breaks less efficiently in response to irradiation and cause a progressive increase in γH2AX (An et al. 2015). TET enzymes also associate with R-loops and DNA:RNA hybrids, which are implicated in genomic instability. Sabino et al. (2022) demonstrated that R-loops and 5hmC patterns correlate genome-wide in mouse and human stem cells and that depletion of TET enzymes reduces both 5hmC and R-loops. Another study using a mouse model of DLBCL showed that deletion of *TET2/3* increased R-loops genome-wide and drove DLBCL oncogenesis (Shukla et al. 2022). *DNMT1* deletion delayed oncogenesis in a TET-deficient background, suggesting cross-talk between TET and DNMT1 in regulating R-loops in DLBCL (Shukla et al. 2022). The complex and seemingly paradoxical nature of this relationship warrants further exploration into the cell type-specific mechanisms controlling R-loops. Since genome instability is a hallmark of cancer (Hanahan 2022), additional work is required to understand exactly how TET enzymes impact DNA damage repair, secondary DNA structures, and other processes impacting genome integrity like DNA replication.

Another key enzyme in DNA demethylation is TDG, which aids in the excision of 5fC and 5caC with base excision repair (BER), resulting in a removal of DNA modifications (Figs. 1, 2; Cortázar et al. 2011; Cortellino et al. 2011; He et al. 2011; Maiti and Drohat 2011). Alterations in *TDG* expression are a biomarker for melanoma and colorectal cancers (CRCs) (Mancuso et al. 2019; Tricarico et al. 2023) and play a role with TET1 in genomic instability through DNA demethylation and inflammation. Loss of TET1 or TDG in CRC cells enhances the inflammatory response and improves tumor killing by NK cells (Tricarico et al. 2023). These epigenetic erasers of DNA methylation show promise as biomarkers and potential therapeutic targets in cancer. For instance, *TDG* is highly expressed in melanoma and, upon depletion, induces cell cycle arrest and senescence, thus inhibiting cell proliferation (Mancuso et al. 2019). Furthermore, *TDG* depletion in vivo reduces tumor growth and is a promising novel target for DNA methylation therapeutic approaches (Mancuso et al. 2019).

### DNA methylation binding protein readers

There are several groups of DNA methylation binding proteins (MBPs) that include methyl-CpG zinc finger proteins, MBD-containing proteins, and SRA domain-containing proteins (Fig. 1; Mahmood and Rabbani 2019). These factors regulate transcription at the DNA methylation modification level by directly binding 5mC, 5hmC, and other cytosine residues. This binding can activate or repress transcription through recruiting additional chromatin-modifying enzymes (Fig. 2; Schübeler 2015). Many of the mechanisms by which DNA methylation modifications are “read” and acted on are still not fully known. Since MBPs are misexpressed or mutated across diseases (Fig. 4), this information will likely provide needed insights about how gene expression programs and genome stability are controlled during physiological processes as well as during pathological states (Mahmood and Rabbani 2019; Mattei et al. 2022; Younesian et al. 2022).

The ubiquitin-like with PHD and ring finger family of enzymes (UHRF1/2) recognizes hemimethylated DNA and recruits its writer component, DNMT1 (Fig. 1; Bostick et al. 2007; Sharif et al. 2007). UHRF1 is an SRA domain-containing protein that is only present in actively proliferating tissue but not in terminally differentiated tissue and is overexpressed in a variety of solid tumors (e.g., osteosarcoma [OS]) (Fig. 4; Mancini et al. 2021). UHRF proteins are implicated in numerous cancer types where they promote migration, proliferation, and metastasis and in silencing tumor suppressor genes (Mahmood and Rabbani 2019). Recently, UHRF1 was identified as a mediator of KRAS-driven lung cancer, whereby knockout of *UHRF1* in *KRAS* mutant cells leads to reduced tumor growth and promotes apoptosis (Kostyrko et al. 2023). Another study in head and neck squamous cell carcinoma (HNSSC) found that SMYD3, a regulator of immune escape in cancer, binds to UHRF1 to suppress transcription of immune genes in vivo (Nigam et al. 2023). For this reason, there are active efforts to therapeutically target UHRF1, which needs further exploration (Myrianthopoulos et al. 2016).

Methyl-CpG binding protein 2 (MeCP2) is another well-known 5mC reader that is involved in several cancers due to its ability to bind to methylated CpG dinucleotides on promoters of tumor suppressor genes and repress gene expression (Fig. 2; Klose et al. 2005). For example, a study in breast cancer cells demonstrated that MeCP2 binds to the promoter regions of *ribosomal protein L11* (*RPL11*) and *ribosomal protein L5* (*RPL5*) genes and reduces their expression, which then promotes a ubiquitin-mediated p53 degradation pathway promoting breast cancer cell growth (Tong et al. 2020). Along with gene suppression, MeCP2 protects cells from genomic instability by preventing R-loops (Marchena-Cruz et al. 2023). In neurological X-linked Rett syndrome (RTT), >90% of the patients have *MeCP2* mutations (Fig. 4; Neul et al. 2008). MeCP2 was shown to bind to 5mC- and 5hmC-containing DNA, which is high in the brain and facilitates gene transcription in neural cell types; however, in RTT, the R133C mutation reduced 5hmC binding by MeCP2, which suggested a shift in binding affinity in the brain (Mellén et al. 2012). Loss-of-function *MeCP2* mutations in human iPSCs and a murine model reduce DNA binding and change chromatin interactions without altering MeCP2 protein levels (Zhou et al. 2023). These data suggest that MBPs could have multiple roles in regulating normal cell function and disease outcome through reading DNA methylation states—not just 5mC, but the other DNA modifications regulated by enzymes such as the TETs. Studies establishing the exact mechanistic underpinning of MBPs and how they are altered by the DNA modification continuum (5mC–5hmC–5fC–5caC) or associated histone modifications will further our insights into their links and cause of disease, which will ultimately provide valuable biomarkers and therapeutic avenues.

## Histone acetylation

### Histone acetylation writers (HATs/KATs)

Histone acetylation promotes a less compacted chromatin state via two primary mechanisms: (1) by generating a differential charge through the negatively charged acetyl group neutralizing the positive lysine and (2) by serving as a platform to facilitate recruitment of coregulatory complexes and RNA polymerase complexes to enhancers and gene promoters (Shvedunova and Akhtar 2022). The following examples highlight the link between KATs, their mechanism(s), and disease.

**Figure 5.**
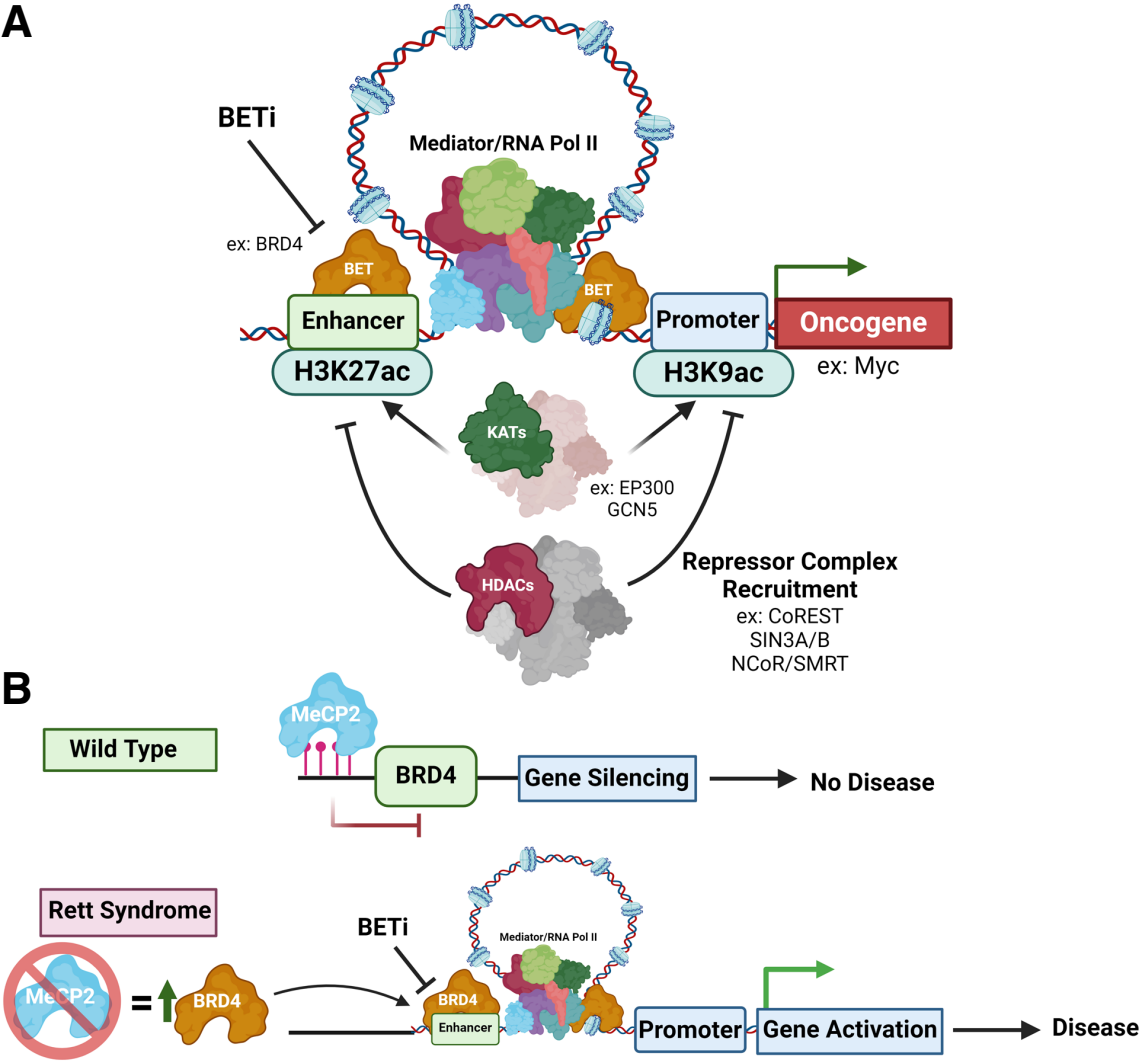
The impact of acetyl writers, erasers, and readers on gene regulation. (*A*) Illustration noting how KATs promote acetylation and BET recognition of acetylation, and in turn promote enhancer–promoter interactions for increased oncogene expression. This activity is countered by HDAC repressor complexes. (*B*) In Rett syndrome, BRD4 is overexpressed and promotes disease phenotypes that can be prevented with BET inhibitors (Xiang et al. 2020).

The E1A binding protein (EP300/P300 or KAT3B) and its paralog, the CREB binding protein (CREBBP, CBP, and KAT3A), catalyze acetylation on multiple lysine residues within histone tails (e.g., H3K27) as well as within other proteins (Zhu et al. 2023b). Moreover, EP300 and CBP are both large proteins with multiple functional domains participating in a variety of protein–protein interactions (Goodman and Smolik 2000). EP300/CBP are involved in numerous cellular processes such as transcription, DNA repair, cell proliferation, and apoptosis and play a fundamental role in enhancer–promoter activity, especially preinitiation complex (PIC) assembly and polymerase pause release (Fig. 5A; Narita et al. 2021). In AML, the activity of EP300/CBP is enhanced through translocations (e.g., *MOZ-CBP* or *MLL/EP300*) (Fig. 4; Miyamoto et al. 2020). Studies have also demonstrated that EP300/CBP shows strong binding affinities at enhancers of oncogenes implicated in hematological malignancies (Zhu et al. 2023b). Treatment of myeloma cells with a potent and selective inhibitor of EP300/CBP, CCS1477 (inobrodib), evicted EP300/CBP from the enhancers of genes, inducing differentiation (Picaud et al. 2015). Preclinical and early-stage clinical studies also demonstrated that disrupting enhancer recruitment of EP300/CBP using inobrodib is a promising therapeutic strategy for myeloid diseases (Nicosia et al. 2023). Consistent with links to cancer, trials occurred or are under way that target EP300/CBP (NCT05488548, NCT03568656, NCT04068597, and NCT04575766) (Armstrong et al. 2021; Eickhoff et al. 2022).

While prior studies focused on EP300/CBP as a unit, a more recent study illustrated that neuroblastoma (NB) cells depend on EP300 but not CBP. Since enhancer control is critical in NB, targeted EP300 degradation resulted in apoptosis (Durbin et al. 2022). These data also illustrate that EP300 and CBP should be considered separately when evaluating tumor function. The development of new compounds capable of degrading or selectively inhibiting EP300 and/or CBP may serve as optimal therapeutics against a range of cancers (Liu et al. 2023).

Genetic alterations and the misregulation of EP300/CBP function are also associated with genome instability, recurrence, and overall survival and multiple types of therapeutic sensitivities, including tamoxifen resistance and gemcitabine sensitivity (Chen et al. 2021; Huang et al. 2021; Luo et al. 2023). For instance, the LIM protein Ajuba plays a crucial role in breast cancer by recruiting deleted in breast cancer-1 (DBC1; also known as CCAR2 or p30DBC) and transcriptional coactivators EP300/CBP to form a tertiary complex. This complex promotes the nonhistone acetylation of estrogen receptor α (ERα) and activates ERα-dependent gene transcription (Xu et al. 2019). Elevated levels of Ajuba not only stimulate breast cancer cell growth but also contribute to tamoxifen resistance, making the Ajuba/DBC1/CBP/EP300 ternary complex a new promising target for breast cancer therapy. It is also essential to consider the role of these writers outside of their effect on chromatin. The effect of CBP/EP300 has been shown to influence cellular metabolism due to the volume of lysine targets on histone tails for acetylation (Weinert et al. 2014, 2018). When considering the effectiveness of these inhibitors, the role of acetylation as a more global regulatory mechanism should also be considered.

Along with EP300/CBP, other KATs are implicated in cancer development and progression (Srivastava et al. 2023) as well as other diseases. For example, the first acetyltransferase discovered, Gcn5 (Brownell et al. 1996), has altered expression or activity and is implicated in a wide range of cancers (Fig. 4; Shao et al. 2018). Gcn5 aids in the progression of cancer through its cooperation with the oncoprotein Myc (Farria et al. 2020), where it acetylates the K323 of Myc and increases Myc stability (Hurd et al. 2023). Gcn5 also drives cell cycle genes through direct histone acetylation, increasing chromatin accessibility and enhancing their expression (Fig. 5A; Liu et al. 2013). *KAT1/HAT1* is also overexpressed and causes an increase in *PD-L1* expression to regulate cancer immunity in pancreatic cancer cell lines and mouse models (Fan et al. 2019). Furthermore, HBO1 (also known as KAT7) (Xiao et al. 2021a) is a major source of histone H3 and H4 acetylation and is involved in crucial cellular processes such as transcription and DNA replication (Xiao et al. 2021a). HBO1 acetylates H3K14, facilitating RNA polymerase II activity to ensure high expression of key leukemia stem cell genes (MacPherson et al. 2020). *HBO1* is also overexpressed in AML (Tsherniak et al. 2017) and in breast cancer (Fig. 4; Ma et al. 2023), making it a plausible therapeutic target in these cancers. Aside from cancer, histone acetyltransferases are implicated in neurodevelopmental disorders such as *TAF1/TAFII250/KAT4* mutations in autism spectrum disorder (ASD) (Gudmundsson et al. 2019), *MYST3/KAT6A* mutations in Arboleda-Tham syndrome (ARTHS) (Tham et al. 2015), and *HRNPA2B1* loss-of-function mutations in amyotrophic lateral sclerosis (ALS) (Fig. 4; Kim et al. 2022). Continued research and mechanistic understanding of histone acetylation writers in combination with their effects on and susceptibility to metabolic states will be required to further define their links to disease.

### Histone acetylation erasers

Histone deacetylases (HDACs or KDACs), are regularly implicated in several pathologies, especially cancer, which prompted the clinical trials and FDA approvals for HDAC therapies (Fig. 4; Ho et al. 2020; Bondarev et al. 2021). The balance of acetylation on histones as well as nonhistone substrates is critical (Shvedunova and Akhtar 2022). HDACs play crucial roles in cancer by deacetylating histone and nonhistone proteins, which are involved in the regulation of transcription, cell cycle, apoptosis, DNA damage response, metastasis, angiogenesis, autophagy, and other cellular processes (Li et al. 2020). For example, multiple HDACs are involved in different stages of cancer such as initiation and progression to metastasis (Li and Seto 2016). Aberrant expression of classical HDACs (classes I, II, and IV) has been linked to a variety of malignancies including solid and hematological cancer (Fig. 4; Kawai et al. 2003; Zhu et al. 2004; Xiong et al. 2019; Singh et al. 2022). In addition, *HDAC* loss-of-function mutations are linked to neurodevelopmental disorders, as observed with *HDAC3* in Huntington's disease (HD) (Jia et al. 2016), *HDAC4* in brachydactyly mental retardation syndrome (BDMR) (Williams et al. 2010), and *HDAC8* in Cornelia de Lange syndrome (CdLS) (Fig. 4; Kaiser et al. 2014). HDACs are commonly associated with multiprotein complexes, specifically NuRD (class I HDACs), CoREST (HDAC1 and HDAC2), SIN3A/B (HDAC1 and HDAC2), and NCoR/SMRT (class IIa HDACs) (Park and Kim 2020), as well as BCoR and CtBP corepressor complexes (Shi et al. 2003; Pagan et al. 2007), which are recruited to chromatin through interactions with numerous transcription factors and other associated proteins in their complexes (Fig. 5A). The data suggest that the formation of these complexes enhances their catalytic activity or specificity. Dysfunction of HDACs/corepressor complexes is believed to cause disruption in gene expression regulation and contribute to disease (Wang et al. 2020). Additionally, a combined multiplex single-cell transcriptomic and chemical screen for HDAC inhibitors demonstrated their varying impact on the regulation of metabolic pathways controlling acetate reservoirs in different cancer cell types (Srivatsan et al. 2020). Therefore, the impact on metabolism and gene regulation is crucial to consider when using HDAC inhibitors for cancer treatment.

Along with cancer, acetylation balance and HDACs have various roles in cardiac pathology, primarily through directly impacting transcriptional programs (Greco and Condorelli 2015; Bagchi and Weeks 2019). Poleshko et al. (2017) revealed the role of HDAC3 in mediating the spatial positioning of lineage-specific loci during cardiogenesis. HDAC3 prevents the differentiation of cardiac progenitor cells and premature expression of cardiomyocyte genes. In embryonic stem cell models, HDAC3 tethers cardiac lineage genes located in lamina-associated domains (LADs) to the nuclear periphery, where the LADs become facultative heterochromatin and enriched in the repressive H3K9me2 modification (Poleshko et al. 2017). Further studying the role of HDACs in spatial positioning of chromatin will shed light on human diseases involving defects in the nuclear lamina such as cardiomyopathies and muscular dystrophies.

Both differential acetylation and HDAC patterns are observed in Alzheimer's disease (AD) patients and may serve as a promising biomarker and potential therapeutic target (Schueller et al. 2020). When treated with trichostatin A (TSA), a pan-inhibitor of HDACs, an APP/PS1 Alzheimer's mouse model showed increased expression of lysosomal and autophagy genes leading to lysosomal biogenesis, improved memory capabilities, and decreased β-amyloid plaque burden (Li et al. 2022a). Another study by Kurita et al. (2012) revealed that in the frontal cortex of schizophrenic patients treated with atypical antipsychotics, the expression of *HDAC2* is elevated, while *HDAC1* and *HDAC4* levels remain unchanged. Moreover, the investigators demonstrated that chronic administration of the antipsychotic clozapine results in increased binding of HDAC2 to the mGluR2 promoter. This clozapine-induced effect is associated with a reduction in histone H3 acetylation at the mGluR2 promoter, subsequently resulting in the downregulation of mGluR2 gene expression in both the human and mouse frontal cortex (Kurita et al. 2012).

### Histone acetylation readers

The most widely studied histone acetylation reader family is the bromodomain and extraterminal domain (BET) family of proteins containing BRD2, BRD3, BRD4, and testis-specific BRDT (Cheung et al. 2021). BET proteins play a wide role in DNA replication, chromatin remodeling, DNA damage, and transcriptional regulation and have been studied extensively in the context of a number of diseases (Cheung et al. 2021). In AML, the bromodomain-containing proteins 3 and 4 (BRD3 and BRD4) are key components of the polymerase-associated factor complex (PAFc) and of the superelongation complex (SEC) (Dawson et al. 2011; Roe et al. 2015). BET inhibition in human and murine MLL cell lines reduces the expression of critical transformation regulators such as MYC, BCL2, and CDK6. BRD3–BRD4 recruit PAFc and SEC to chromatin and recruit RNA polymerase II to the promoters of these oncogenes. BET proteins also localize to superenhancers (SEs) of pathology-associated genes and promote their expression (Fig. 5A,B; Chapuy et al. 2013; Hnisz et al. 2013; Roe et al. 2015; Sabari et al. 2018). Furthermore, these studies pointed to an essential role of BET proteins in SE-driven *MYC* expression that promotes proliferation and cell survival in neuroblastoma and diffuse intrinsic pontine glioma (Chipumuro et al. 2014; Nagaraja et al. 2017). These studies and others led to efforts to chemically target BET proteins and the BRDs and have resulted in numerous clinical trials (Shorstova et al. 2021).

Studies have highlighted highly distinct functions for individual BET proteins across diseases. For example, CBP/p300 depletion in a fragile X syndrome (FXS) mouse model resulted in decreased binding of BRD4 but not BRD2/3 to promoters and enhancers of regulatory regions and rescued behavioral impairments (Kim et al. 2021). Additionally, studies in Rett syndrome (RTT) models found that a loss of methyl-CpG binding protein 2 (MeCP2) led to an increase of *BRD4* expression (Fig. 5B). Treatment with the pan-BET inhibitor JQ1, initially described by Bradner and colleagues (Filippakopoulos et al. 2010), reduced transcriptional hyperactivation in MeCP2-mutated human cortical interneurons (Xiang et al. 2020). Treatment of an MeCP2-null (MeCP^−/Y^) mouse model with JQ1 resulted in extended life span and reduced RTT symptoms (Fig. 5B; Xiang et al. 2020). Reports have also shown distinct activities between different isoforms of BET proteins (Wu et al. 2020), suggesting that specific targeting of each of these proteins will be optimal for addressing diseases in the future.

Another emerging reader family impacting acetylation function consists of the YEATS domains. The YEATS domain recognizes histone acetylation, resides within various chromatin-modifying enzymes, and plays biological roles in transcriptional elongation and chromatin modifications and remodeling. There are four subtypes of proteins containing this domain, including AF9, ENL, glioma-amplified sequence 41 (GAS41), and YEATS2. In non-small-cell lung cancers (NSCLC), GAS41 is frequently upregulated and promotes cancer cell proliferation and survival through promoting histone variant H2A.Z deposition (Fig. 4; Hsu et al. 2018). GAS41 localizes on the promoters of active genes through binding H3K27ac and H3K14ac. Depletion or disruption of GAS41 or disruption of the interaction between its YEATS domain and acetylated histones impairs the deposition of H2A.Z with chromatin, suppressing cancer cell growth and survival in vivo (Hsu et al. 2018). Interestingly, two of the YEATS-containing proteins, AF9 and ENL, are commonly rearranged and have been implicated in driving leukemia (Figs. 1, 4). Recent studies identified a small molecule inhibitor targeting the YEATS domain interaction of ENL/AF9 but not GAS41 or YEATS2. This inhibitor, TDI-11055, completely blocked disease progression in *MLL-*rearranged and *NPM1-*mutated leukemia models through disrupting key oncogenic transcriptional programs that these tumors depend on (Liu et al. 2022). Future studies should focus on developing such inhibitors in order to minimize the toxicity and off-target effects.

## Histone lysine methylation

### Histone lysine methylation writers

The temporal and spatial coordination of histone lysine methylation and the degree of methylation (monomethylation [Kme1], dimethylation [Kme2], and trimethylation [Kme3]) regulate most DNA templated processes (Husmann and Gozani 2019). Therefore, various pathologies exhibit mutations, genetic translocations, and/or altered expression of the lysine methyl writers, lysine methyltransferases (KMTs) (Fig. 4; Husmann and Gozani 2019). For this reason, the field is actively characterizing their function and establishing their potential as biomarkers and therapeutic targets. Some examples are highlighted below.

**Figure 6.**
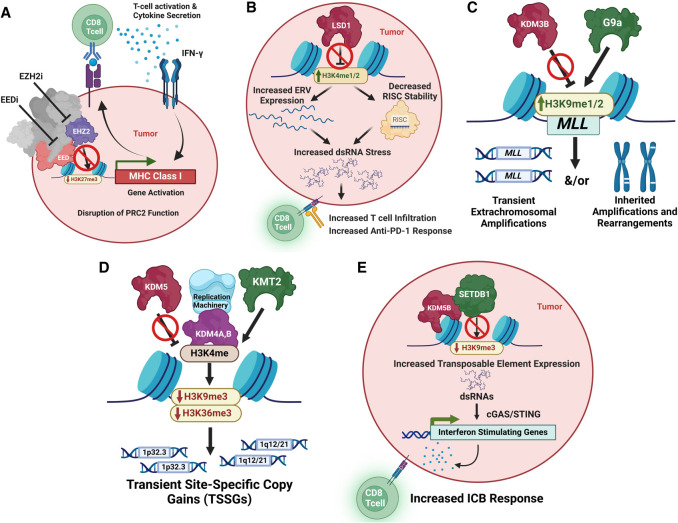
Histone lysine methylation writer, reader, and eraser balance and the regulation of transcription and DNA amplification. (*A*) Illustration showing how PRC2/EZH2/EED repress MHC class I genes and, upon inhibition, promote immune responses in tumors (Burr et al. 2019). (*B*) Illustration highlighting how KDM1A/LSD1 inhibition can be used to promote increased immune activation in tumors (Sheng et al. 2018, 2021; Liu et al. 2021). (*C*) Schematic showing the balance between KDM3B and G9a in regulating MLL ecDNA amplifications and rearrangements (Gray et al. 2023). (*D*) Illustration highlighting the interplay between KMT2, KDM4, and KDM5 family members in regulating transient site-specific copy gains (TSSGs) (Mishra et al. 2018; Clarke et al. 2020). (*E*) Schematic illustrating how KDM5B recruits SETDB1 so that proper repression of retroviral elements occurs, which provides an opportunity to increase their expression and in turn enhances ICB by inhibiting KDM5B and SETDB1 (Zhang et al. 2021c).

In the context of cell function and disease, there has been a significant focus on the main H3K27me3 KMT, EZH2 (Fig. 1). EZH2 is the enzymatic subunit of the *polycomb-repressive complex 2* (*PRC2*) that is overexpressed or has gain-of-function mutations driving methylation in multiple cancers (Fig. 4; Schuettengruber et al. 2017). EZH2 harbors critical functions through gene silencing by trimethylation of H3K27, which impacts cell fate, cell cycle progression, autophagy, apoptosis, DNA damage repair, and cellular senescence and plays a role in lineage determination (Schuettengruber et al. 2017; Batool et al. 2019). EZH2/PRC2 also has a conserved role in modulating MHC-I antigen presentation, which allows cancer cells to evade immune cells; therefore, EZH2 inhibition or inhibition of embryonic ectoderm development (EED; a reader in the PRC2 complex) (see below; Fig. 1) promotes MHC-I expression and antitumor immunity (Fig. 6A; Burr et al. 2019). In mice, EZH2 inhibition increases the activity of NK cells and T-cells within the tumor microenvironment, leading to improved overall survival (Chibaya et al. 2023). Immune suppression driven by EZH2 is also observed in bladder cancer, where catalytic inhibition improves T-cell infiltration and suppresses tumor progression. Mice lacking an intact immune system experience no antitumor effects upon EZH2 inhibition (Piunti et al. 2022). Together, these data highlight a potential role for EZH2 in tumor immunity, making this a critical area to investigate.

In addition to being a repressor of transcription, studies illustrate that EZH2 alone can promote transcription that is independent of PRC2 and methylation activity in prostate cancer (Kim et al. 2018). EZH2 directly activates *androgen receptor* (*AR*) gene transcription by occupying its promoter (Kim et al. 2018). In liquid tumors, EZH2 creates a noncanonical complex with Myc (MYCN or cMyc) and EP300, binding at non-PRC2 targets and activating their transcription (Vanden Bempt et al. 2022; Wang et al. 2022). While EZH2 enzymatic activity was not required for this activation, loss of EZH2 using degraders prevented transcriptional activation (Wang et al. 2022; Yu et al. 2023). These data underscore the importance of the noncatalytic, noncanonical activities of KMTs and the importance of understanding their associated complexes and their contribution to gene modulation and disease progression.

EZH2 inhibitors are in clinical development (Simon and Lange 2008; Italiano et al. 2018; Paskeh et al. 2022). Tazemetostat is the most advanced EZH2 inhibitor and was granted accelerated approval for adults with relapsed or refractory (R/R) follicular lymphoma (FL) whose tumors are positive for an *EZH2* mutation (Fig. 4; Italiano et al. 2018). Up to 25% of all DLBCL and FL cancers contain gain-of-function heterozygous mutations in the SET domain of *EZH2* at tyrosine 641 (Y641) (Fig. 4; Morin et al. 2010). Studies also demonstrate a coordinated methylation activity between the WT and mutant alleles in these tumors, with the WT *EZH2* allele preferentially placing monomethylation, and the mutant allele placing dimethylation and trimethylation. Therefore, the widespread redistribution of H3K27me3 in these tumors is likely due to the coordination of the two alleles (Sneeringer et al. 2010; Souroullas et al. 2024). Other EZH2 inhibitors are in clinical trials, including valemetostat, CPI-1205, and CPI-0209 for R/R adult T-cell leukemia/lymphoma (Izutsu et al. 2023) and metastatic castration-resistant prostate cancer (NCT04846478) (Liu and Yang 2023). A recent study reported that a novel EZH2 inhibitor (GSK126) (Yap et al. 2019) opened the condensed and H3K27me3-marked chromatin in AML cells. Use of this inhibitor enhanced DNA damage and apoptosis induced by chemotherapeutic agents, including the topoisomerase II inhibitors doxorubicin and etoposide (Porazzi et al. 2022). These data emphasize the need to consider targeting DNA damage and repair in conjunction with EZH2 inhibition.

Along with EZH2, other KMTs are dysregulated in cancer and other diseases (Fig. 4). For example, *nuclear receptor SET domain* (*NSD*)-containing KMTs are mutated and hyperactivated in hematologic and solid malignancies (Bennett et al. 2017). In lung adenocarcinoma, for example, there are activating mutations in the H3K36me2 KMT *NSD2/MMSET* (Fig. 4). Genetic depletion of *NSD2* leads to decreased *VEGF* expression and reduced tumor growth (Sengupta et al. 2021). *NSD2/MMSET* also has an activating translocation in 20% of multiple myeloma (MM) cases (Fig. 4; Kuo et al. 2011). *NSD2* activation enhanced the rate of DNA damage repair, which contributed to chemotherapeutic resistance in MM (Shah et al. 2016). There is also emerging evidence that NSD2 plays a direct role in genome organization and stability (Lhoumaud et al. 2019). *NSD2* fusions in MM led to spreading of H3K36me2 into intergenic regions and altered H3K27ac, which promoted gene expression dysregulation and genome compartment switching (Lhoumaud et al. 2019). These links to cancer and genome regulation have prompted therapeutic development, including the NSD2 inhibitor KTX-1001 that is currently being evaluated in the treatment of R/R MM (NCT05651932).

The *MLL* genes (*MLL1–4*) encode for proteins that catalyze H3K4 methylation. These genes are a recurrent site of genetic alterations, including rearrangements, linked to cancer (Fig. 4; Mohan et al. 2010; Husmann and Gozani 2019). Translocations involving *MLL1* (*MLL/KMT2A*) can generate fusions with SEC members, which is required for *MLL* chimera target gene activation through the misregulation of transcription elongation checkpoint control (Fig. 4; Luo et al. 2012). The SEC is recruited to specific genomic loci and causes rapid transcriptional induction (Luo et al. 2012). Furthermore, MLL3 and MLL4 form multiprotein complexes regulating H3K4me1 at enhancers and work together with EP300/CBP-mediated H3K27 acetylation to generate the active enhancer landscape for long-range gene activation (Herz et al. 2014; Morgan and Shilatifard 2015). *MLL3/4* (*KMT2C/D*) are frequently mutated and thought to promote cancer through enhancer malfunction (Fig. 4; Herz et al. 2014; Lawrence et al. 2014; Morgan and Shilatifard 2015). While some mutations are inactivating, it is also important to consider the fact that these proteins (like most epigenetic factors) are part of large complexes; therefore, altered consequences could emerge (Morgan and Shilatifard 2020).

Another critical KMT associated with hematologic malignancies is the H3K79 KMT, disruptor of telomeric silencing 1-like (DOT1L) (Lacoste et al. 2002; van Leeuwen et al. 2002; Okada et al. 2005; Alexandrova et al. 2022; Yi and Ge 2022). MLL chimeras recruit DOT1L to inappropriate sites and promote gene expression (Okada et al. 2005). DOT1L inhibitors have been leveraged against pediatric and adult leukemias with *MLL* rearrangements. In the clinic, there has been modest activity in improving therapeutics for *MLL* rearrangement-driven adult acute leukemia patients (Stein et al. 2018). In an attempt to target the *MLL*-driven cancers, inhibitors to the adapter protein Menin have been developed. Menin is a scaffolding protein that is known to have both positive and negative functions contributing to transcription and cell signaling in disease showing promising targeting for AML treatment (Swaminathan et al. 2022). In fact, Issa et al. (2023) report in a phase 1 clinical trial that revumenib (SNDX-5613), a menin-MLL inhibitor, resulted in partial or complete remission in patients with relapsed or refractory NPM-1-mutated leukemia. Therefore, a clear strategy to consider is targeting accessory proteins that facilitate key functions promoting oncogenesis. This area is important for future biomarker and drug development.

In lung cancer, gain-of-function mutations in the catalytic domain of DOT1L promote malignant phenotypes via the MAPK/ERK signaling pathway (Fig. 4; Zhang et al. 2023). This study identified three common mutations in the DOT catalytic domain of DOT1L (E186A, S225L, and R231Q), all of which had higher levels of H3K79me2 in their corresponding cell lines. The variant with the strongest effect on H3K79me2, R231Q, enhances the substrate binding ability of DOT1L and promotes cell growth and drug resistance of lung cancer cells in vitro and in vivo (Zhang et al. 2023). Mechanistic characterization of this variant also showed that R231Q specifically activates the MAPK/ERK signaling pathway by enriching H3K79me2 on the RAF1 promoter and epigenetically regulates the expression of downstream targets such as *LK3* and *KLF4*.

While the examples above link KMTs to cancer, their dysregulation impacts other diseases (e.g., cardiac and neurodevelopmental diseases) (Fig. 4; Van Rechem and Whetstine 2014; Zhu et al. 2023a). *KMT2D/MLL4* mutations are associated with Kabuki syndrome, a rare congenital disease that presents with congenital heart defects in >70% of patients (Fig. 4; Digilio et al. 2017). Haploinsufficiency of KMTs is also found in several developmental disorders, including Sotos syndrome (NSD1) (Kurotaki et al. 2002), Beckwith-Wiedemann syndrome (NSD1) (Baujat et al. 2004), and Weaver syndrome (EZH2) (Fig. 4; Gibson et al. 2012). In addition to correlative genetic studies in regards to KMTs in developmental diseases, large-scale genetic screening identified KMT5B (SUV420H1) in autism spectrum disorder (ASD) (Fig. 4; Wang et al. 2021). KMT5B deficiency in the prefrontal cortex (PFC) induces transcriptional changes, alters DNA repair, impairs glutamatergic transmission, and induces social deficits (Wang et al. 2021). These findings provide a framework for understanding the molecular mechanisms that link KMT haploinsufficiency and ASD. With the emerging information in oncology and other disease models, the field will be able to better understand the impact that KMT mutations and genetic alterations have on disease. The collective knowledge will advance mechanistic insights while building ways to diagnose and treat disease most appropriately.

### Histone lysine methylation erasers

Histone lysine demethylase (KDM) eraser enzymes have also been implicated in multiple diseases (Fig. 4). For example, *KDM1A/LSD1*, a H3K4me1/2 demethylase, is overexpressed in a variety of cancers including breast (Zhou et al. 2021), small cell lung cancer (Jin et al. 2019), and AML (Fig. 4; Zhang et al. 2021b). *LSD1* overexpression correlates with poor patient survival (Hayami et al. 2011) and promotes cancer through various mechanisms. For example, LSD1 prevents PHD2-induced hydroxylation and enhances K532 deacetylation of HIF-1α, which drives breast cancer (Lee et al. 2017). LSD1 also suppresses antitumor immunity in tumors, while LSD1 inhibition promotes immunotherapy response (Fig. 6B; Sheng et al. 2018, 2021; Liu et al. 2021). In fact, LSD1 stabilizes a component of the RISC, AGO2, through demethylation of K276. LSD1 inhibition leads to decreased protein levels of AGO2, leading to dsRNA stress and IFN activation in cancer cells (Sheng et al. 2018). In leukemia, LSD1 may play a critical role through suppressing myeloid differentiation (Nicosia et al. 2022). LSD1 binds to the chromatin protein GSE1, localizing at promoters to enforce transcriptional silencing and suppressing myeloid differentiation. Inhibition of LSD1 in AML models suppresses the GSE1–LSD1 interaction, rescuing the transcriptional programs and promoting differentiation (Nicosia et al. 2022). These studies suggest a therapeutic opportunity alone or in combination with current immune checkpoint therapies to combat these mechanisms. To date, nine LSD1 inhibitors are being evaluated in clinical trials, with more in the pipeline (Noce et al. 2023).

The JmjC family of demethylases has also been widely implicated in cancer. For example, KDM2B is a catalytic member of the polycomb-repressive complex 1 (PRC1), which is implicated in a variety of cancers (Fig. 4; Yan et al. 2018). In contrast, KDM3B is considered a tumor suppressor (Hu et al. 2001). *KDM3B* is commonly deleted and underexpressed in MDS and AML and correlates with poor prognosis (Fig. 4; Xu et al. 2018). KMD3B loss or chemical inhibition directly promotes *MLL/KMT2A* extrachromosomal amplifications and rearrangements, which are often associated with *KMD3B* LOH in AML (Gray et al. 2023). *MLL/KMT2A* alterations are generated through topoisomerase inhibitor therapies, and these inhibitors phenocopy tumors in that they reduce KDM3B protein levels. The reduced KDM3B causes these *MLL* alterations, which can be blocked with EHMT2/G9a methyltransferase inhibition, highlighting a novel method to prevent *MLL* alterations upon chemotherapy treatment (Fig. 6C; Gray et al. 2023). The importance of balancing methylation for extrachromosomal DNA (ecDNA) amplification control was also shown to be the case for the KDM4 (H3K9/36me3/2 KDMs) and KDM5 (H3K4me3 KDMs) families of enzymes (Mishra et al. 2018; Clarke et al. 2020). KDM4 and KDM5 inhibitors were used to effectively oscillate the ecDNAs in noncancer and cancer cells, which illustrated the importance of epigenetic regulation in controlling ecDNAs (Fig. 6D; Mishra et al. 2018; Clarke et al. 2020).

Along with regulating DNA replication and amplification (Black et al. 2010, 2013; Wu et al. 2017; Van Rechem et al. 2021), the KDM4 family members impact cancer through several other avenues. *KDM4A* is amplified and overexpressed in multiple cancers, including ovarian, breast, prostate, and lymphoma (Fig. 4; Black et al. 2013). KDM4A is also stabilized in hypoxic environments, a key feature of tumors (Black et al. 2015). In lung cancer, KDM4A collaborates with oncogenic KRAS to promote cellular transformation by downregulating tumor suppressor chromodomain helicase DNA binding protein 5 (CHD5) (Mallette and Richard 2012). KDM4A also drives leukemogenesis through promoting self-renewal and survival of AML cells via the KDM4A–PAF1 signaling-mediated transcriptional program (Filiú-Braga et al. 2019). Furthermore, a polymorphism in *KDM4A* is associated with differential NSCLC outcomes (Van Rechem et al. 2015b). This variant impacted KDM4A stability and in turn sensitivity to therapies associated with translation like mTOR inhibitors (Van Rechem et al. 2015a,b). The use of a KDM4 inhibitor (JIB-04) also sensitizes cells to mTOR inhibitors (Van Rechem et al. 2015a). Currently, there are four classes of KDM4 inhibitors depending on their mechanism: (1) α-KG and 2-OG cofactor mimics (Itoh et al. 2013), (2) metal cofactor disruptors (Giri et al. 2013), (3) histone substrate competitive inhibitors (Wang et al. 2013), and (4) noncofactor/nonsubstrate inhibitors (Leurs et al. 2014). TACH101, a KDM4 family inhibitor (Chandhasin et al. 2023), is in phase I clinical trials against advanced or metastatic solid tumors (NCT05076552). The results of this trial will reveal the impact of single-agent activity. However, it seems likely that proper drug combinations will be required to see the full impact of this family of inhibitors based on the recent discoveries.

The H3K4 demethylase enzymes KDM5A and KDM5B are highly expressed in a small population of drug-tolerant cells across multiple cancers (Fig. 4; Roesch et al. 2010; Sharma et al. 2010). Tumors with high expression of KDM5 enzymes contain significantly increased self-renewal capability due to upregulated mitochondrial activity and enhanced gene expression (Ren et al. 2022). KDM5B was shown to directly recruit SETDB1 (H3K9me3 KMT) and silence the endogenous retroviral elements so that tumors were invisible to the immune system (similar to how increased 5mC silences these elements) (Fig. 2; (Okano et al. 1999). This study identified the importance of targeting KDM5B and SETDB1 in order to overcome immune therapy resistance (Fig. 6E). By a different mechanism, KDM4A inhibition impaired DNA replication and increased DNA fragments that trigger the cGAS–STING signaling and antitumor immunity (Zhang et al. 2021c). Collectively, these studies emphasize the benefit of regulating KDM4/5 enzymes but reiterate the interplay and considerations for their impact on genome instability, heterogeneity, immune checkpoint, and drug resistance.

Another KDM mutated and significantly associated with cancer and other diseases is the H3K27me3-specific KDM6 family (Hua et al. 2021). *KDM6A/UTX* is mutated in Kabuki syndrome (Fig. 4; Van Laarhoven et al. 2015). KDM6A also is associated with the MLL complexes impacting gene regulation and associates with multiple cancers (Schulz et al. 2019). In neuroblastoma, KDM6B is highly expressed and transcriptionally activates the oncogenic CDK4/6–pRB–E2F pathway involved in cell cycle regulation (Fig. 4; D'Oto et al. 2021). In AML, KDM6 enzymes play a critical role in regulating the DNA damage response. KDM6A/B loss compromised the DDR potential of AML cells, sensitizing them to PARP inhibitor therapy (Boila et al. 2023). These findings suggest that KDM6 is a promising therapeutic target in these cancers. However, in relapsed AML, KDM6A LOF mutation or reduced expression provides significantly enhanced tumor growth or drug resistance after treatment (Stief et al. 2020). These data highlight that the time line of disease could also be key to understanding when to target these or other epigenetic modulators (e.g., early stage vs. resistant).

Multiple KDMs have been implicated in neurodegenerative disorders including Alzheimer's disease (AD) and Huntington's disease (HD) (Fig. 4). In a mouse model of AD, loss of *LSD1/KDM1A* was found to drive hippocampus and cortex degeneration as well as behavioral decline in memory and learning (Christopher et al. 2017). This study demonstrated that reduction in LSD1 exaggerated tau-mediated neurodegeneration and gene expression changes. When overexpressed, LSD1 promoted a delay in neurodegeneration and gene expression alterations (Christopher et al. 2017). These data highlight the importance of LSD1; however, inhibition studies strongly suggest that inactivation provides benefit to CNS-related diseases (Li et al. 2022b). Therefore, LSD1 inhibition is being targeted in CNS disease (Fang et al. 2019; Antonijoan et al. 2021; Li et al. 2022b). The observed LSD1 paradox for the CNS likely reflects the impact of LSD1 on the cell of origin and the need for LSD1 protein versus enzyme activity. These areas need further understanding in order to properly target LSD1 in CNS-related disease. After all, inhibition could benefit cancer and CNS diseases, while protein destabilization could pose issues long term if LSD1 protein loss promotes CNS defects.

Along with LSD1 impacting the CNS, KDM5C/SMCX/Jarid1c, a H3K4me2/3 demethylase, has been linked to intellectual disabilities, autism, and Huntington's disease (Fig. 4; Lan et al. 2007a; Vashishtha et al. 2013; Vallianatos and Iwase 2015; Iwase et al. 2016; Vallianatos et al. 2018). Furthermore, KDM6A, an H3K27me2/3 demethylase, is associated with X-linked Kabuki syndrome (Fig. 4; Van Laarhoven et al. 2015) and has also been shown to rescue huntingtin-induced HD development in *Drosophila* models (Song et al. 2018). Together, these results suggest that the proper balance of KDMs can promote one disease state or another. Therefore, future efforts need to consider the acute targeting and benefits versus the possible long-term consequences. Collectively, the discoveries surrounding KDMs suggest that they will become biomarkers and therapeutic targets for a host of pathologies in years ahead.

### Histone lysine methylation readers

Many protein domains read lysine methylation states on histones tails (Musselman et al. 2012). For example, Tudor domain-containing proteins are readers that recognize H3K4me3, H3K9me2, H3K36me3, and H4K20me3 (Fig. 1). Tudor readers are involved in physiological processes such as DNA methylation, transcription, DNA damage repair, and rRNA gene expression (Ortiz et al. 2023). The KDM4A-C enzymes contain double-Tudor domains (DTD) that recognize H3K4me3. In order for KDM4A–B to drive site-specific rereplication and ecDNA amplification, their DTDs need to be intact (Mishra et al. 2018). The Tudor domain in the PHF20L1 protein was identified as a promising target in breast cancer. PHF20L1 recognizes H3K27me2 via its Tudor domain and in turn recruits PRC2 complexes to specific tumor suppressors including *BRCA1* (Hou et al. 2020). These examples suggest that targeting Tudor domains with small molecules could be a promising therapy in the future. Work has already begun to identify preliminary hits for small molecules targeting Tudor domains (Mader et al. 2019).

In addition to Tudor domains, there are proline–tryptophan–tryptophan–proline (PWWP) domains that serve as chromatin-reading modules and impact tumorigenesis. Diffuse intrinsic pontine glioma (DIPG), a deadly pediatric brain tumor, is characterized by a point mutation leading to the oncohistone H3K27M that reshapes the epigenome through a global inhibition of PRC2 catalytic activity and displacement of H3K27me2/3 (Chan et al. 2013; Lewis et al. 2013; Nacev et al. 2019). Consequently, H3K36me2 is aberrantly elevated and read by PWWP domain-containing proteins LEDGF and HDGF2, leading to protumorigenic effects of H3K36me2 (LeRoy et al. 2019). Treatment of DIPG cells with a chemically modified peptide mimicking endogenous H3K36me2 dislodges LEDGF and HDGF2 and specifically inhibits H3K27M-DIPG (Yu et al. 2021). Furthermore, the PWWP domain in NSD2 binds H3K36me2/3 and has become another way to target this enzyme in MM (Dilworth et al. 2022). While much focus of reader proteins has been on reading of monomethylation, dimethylation, or trimethylation, there are also reader proteins that recognize unmethylated histone tails (BHC80) (Lan et al. 2007b) and specific histone variants such as H3.1 compared with H3.3 (ZMYND11) (Wen et al. 2014). Collectively, these examples emphasize the degree of specific reading and targeting that occurs in the genome and the need to consider this when leveraging reader therapies.

Another common histone methylation reader domain that exists in a large family of proteins is plant homeodomain (PHD) fingers. PHD fingers can also recognize nonhistone proteins and DNA, which significantly expands their role in the regulation of cellular processes (Gaurav and Kutateladze 2023). Here, we focus on the ability to recognize different histone marks. One example is BHC80, a PHD finger-containing protein of the LSD1 corepressor complex that binds unmethylated histone tails through its PHD finger (Lan et al. 2007b). This recognition is thought to promote recruitment to areas with less methylation. Another key protein involved in targeting a repressor complex is PHD finger protein 1 (PHF1). Expression of *PHF1* is upregulated in human cancers and promotes proliferation and invasion of breast cancer cells (Liu et al. 2018). PHF1 is an accessory member of the polycomb-repressive complex 2 (PRC2) that contains two different kinds of histone reader domains: a Tudor domain and two PHD fingers (Boulay et al. 2011; Schuettengruber et al. 2017). The Tudor domain binds histone marks such as H3K36me3, leading to increased nucleosome accessibility (Musselman et al. 2013), while the N-terminal PHD finger of PHF1 recognizes symmetric demethylation of H4R3 (Liu et al. 2018). Along with accessory proteins being critical for PRC2 targeting, the PRC2 core complex contains the H3K27me3 reader EED, which partakes in a positive feedback mechanism where it recognizes H3K27me3 through its WD40 domain and through allosteric activation where it activates EZH2 to repress gene expression (Hansen et al. 2008; Margueron et al. 2009). In cancer, alterations in *EED* disrupt this feedback mechanism (Imagawa et al. 2017; Lee et al. 2018). *EED* is overexpressed in several solid tumors and leads to increased PRC2 activity (Fig. 4; Veneti et al. 2017). These studies make EED and its WD40 reader domain good therapeutic targets to disrupt the repressive function of EZH2 in PRC2, which is under investigation (Dong et al. 2019).

Another common methyl-reader domain impacting disease is the chromodomain (CD) (Fig. 1). This domain occurs in a host of proteins. The additional functional domains in the CD-containing proteins determine the families: the heterochromatin (HP1)/polycomb (Pc) family, the chromo-ATPase/helicase–DNA binding (CHD) family, the chromobarrel domain family, and the chromodomain Y chromosome (CDY) family (Eissenberg 2012). In humans, HP1 homologs (CBX1, CBX3, and CBX5) play a crucial role in heterochromatin assembly and gene silencing, with a particular affinity for the H3K9me mark (Schalch et al. 2009). In contrast, human Pc homologs (CBX2, CBX4, CBX6, CBX7, and CBX8) bind to both H3K9me3 and H3K27me3 marks (Vermeulen et al. 2010). The chromodomain helicase DNA binding (CHD) proteins read and/or interpret histone modifications using specialized domains to modulate these epigenetic signals. These proteins are essential for establishing and sustaining compact chromatin structures, which are crucial for transcriptional repression and maintaining genomic integrity (Yap and Zhou 2010). CHDs modulate the physical state of chromatin by altering DNA–histone interactions via shifting nucleosomes along DNA (Fig. 3; Mashtalir et al. 2018).

CD-containing proteins are also known for their capacity to promote nuclear condensates. Several CD proteins are known to participate in liquid–liquid phase separation (LLPS), where biomolecules separate into distinct liquid-like compartments within the cytoplasm or nucleus. One of the most prominent examples is oligomerization of the repressive factor HP1a/α (Larson et al. 2017). This process involves both structured and intrinsically disordered regions of HP1 (CD; chromoshadow domain [CSD], and N-terminal extension or its hinge region) (Larson et al. 2017; Sanulli and NarlikarG 2020). HP1a/α has been observed to undergo LLPS while bound to chromatin, suggesting that phase separation may facilitate the organization of constitutive heterochromatin and the selective exclusion of specific proteins from heterochromatin condensates (Larson et al. 2017). These condensates can sequester other proteins and RNAs, thereby modulating their availability for numerous cellular functions (Larson et al. 2017). Notably, PRC1 complexes can form structures known as Polycomb bodies (Xu et al. 2021). These observations are pivotal, as they link alterations in chromatin architecture with cellular processes, proposing that LLPS might serve as a regulatory mechanism to organize and maintain chromatin domains. The broad implications of these findings highlight the complexity of nuclear organization and the potential for targeting CD proteins in therapeutic strategies where 3D partitioning is misregulated.

## Remodelers and their mechanistic link to disease

The four known families of remodelers are the mSWI/SNF, INO80, imitation switch (ISWI), and chromodomain helicase DNA binding (CHD) families (Clapier et al. 2017). These remodeler families are considered derivatives of the SNF2 ATPase family that require ATP to alter histone/DNA contacts. Each remodeling complex contains a catalytic ATPase enzyme that uses ATP hydrolysis to modify chromatin (Figs. 1, 4; Clapier et al. 2017). Through this function, chromatin remodelers regulate multiple cellular processes including transcription, DNA repair, and replication (Tyagi et al. 2016). Here, we discuss examples for remodeler enzyme function and their implications in disease.

The remodelers most implicated in disease are the mSWI/SNF complexes, which influence chromatin architecture and gene expression (Fig. 3; Kadoch and Crabtree 2015; Mittal and Roberts 2020). The main complexes are canonical BRG1/BRM-associated factor (cBAF), polybromo-associated BAF (PBAF), and noncanonical BAF (ncBAF) (Mittal and Roberts 2020), which are composed of multiple subunits, some overlapping between complexes. The composition of these complexes is dynamic during development and is subject to misregulation contributing to multiple disease types. In fact, these complexes undergo alterations in both shared and specific subunits in a variety of human malignancies including in >40% of cancers (Fig. 4; Son and Crabtree 2014; Kadoch and Crabtree 2015; Sima et al. 2019). The main mechanisms of mSWI/SNF mutations in cancer are enhancer dysregulation and oncogene activation (Jones et al. 2022). One of the first documented implications of remodelers in cancer was the identification of BAF as a tumor suppressor in rare malignant rhaboid tumor (MRT) caused by biallelic inactivation of BAF47 (*hSNF5*, *INI1*, and *SMARCB1*) (Versteege et al. 1998; Kadoch and Crabtree 2015). The conditional deletion of BAF47 in mice was found to lead to T-cell lymphomas with a short latency that is unprecedented for the deletion of a single gene (Wang et al. 2011). Furthermore, BAF complexes in the mutant cells were unable to remove Polycomb complexes and their histone modification, H3K27me3, from the Ink4a (*Cdkn2a*) locus, which normally suppresses proliferation (Wilson et al. 2010). Another frequently mutated mSWI/SNF family protein, ATRX, is linked to cancer and a neurodevelopmental disease known as ATRX syndrome (Fig. 4; Pang et al. 2023). Thus, mSWI/SNF complex subunits are of great clinical interest to target in multiple disease types.

In over half of all prostate cancers, chromosomal rearrangements resulting in the fusion of *TMPRSS2*, an androgen-regulated gene, and the ETS family transcription factor *ERG* occur (Sandoval et al. 2018). ERG is known to promote oncogenic gene expression and proliferation through the interaction with SWI/SNF and retargets BAF complexes to ETS DNA motifs. BAF complexes are required for ERG-mediated basal-to-luminal transition in prostate cancer organoids, a known hallmark of ERG activity in prostate cancer (Sandoval et al. 2018). In squamous cell carcinoma (SCC), the BAF subunit actin-like 6a (ACTL6A) is amplified early in the development of SCC. The overexpression of ACTL6A leads to BAF complex assembly and interactions with regulatory areas of the genome that enhanced polycomb redistribition genome-wide (Chang et al. 2021). In the context of therapeutic resistance across cancer types, the ACTL6A subunit is found to be frequently amplified (Vaicekauskaitė et al. 2022). One study identified a role of ACTL6A in repairing cisplatin-induced DNA damage seen in cancer therapeutic resistance. They demonstrated that ACTL6A overexpression promoted repair of cisplatin-induced damage through SWI/SNF in ovarian cancer cells (Xiao et al. 2021b). Additionally, a HDAC inhibitor was shown to prevent cisplatin resistance caused by ACT6A overexpression in a mouse xenograft model. This study unveils additional mechanistic opportunities to combat therapeutic resistance (Fig. 3B; Xiao et al. 2021b). ARID1A is also of interest in therapeutic resistance and prognosis with anti-EGFR cetuximab but not anti-VEGF bevacizumab treatment therapies in colorectal cancer (Johnson et al. 2022). ARID1A mutational status can be used when planning cetuximab treatment to reduce therapeutic resistance and improve patient outcomes (Johnson et al. 2022). In the future, additional links between chromatin remodeler subunits or subfamily classes and therapeutic resistance will likely be revealed due to their involvement in gene regulation, DNA damage repair mechanisms, and nucleosome translocations.

Therapeutic strategies leveraging DNA-damaging drugs such as PARP and ATR inhibitors to induce synthetic lethality in ARID1A-mutated cancers have emerged (Shen et al. 2015; Park et al. 2019). This lethality resulted from nonhomologous end-joining (NHEJ) repair dysregulation. Moreover, the absence of ARID1A results in impaired checkpoint and DNA double-strand break (DSB) repair. This makes cells susceptible to treatments that induce DSBs, such as radiation and PARP inhibitors (Shen et al. 2015; Yakovlev et al. 2023). While the subunit ARID1A is mutated in several tumor types (Fig. 4), it lacks druggable domains. This issue prompted the consideration of EZH2 inhibitors, as they are used to induce synthetic lethality in mutated tumors (Bitler et al. 2015). However, a subunit switch from SMARCA4 to SMARCA2 led to EZH2 inhibitor resistance. A combination approach involving inhibition of the antiapoptotic gene BCL2 due to loss of SMARCA4 leading to suppression of apoptotic pathways was used to combat these effects (Wu et al. 2018). Both direct targeting of these complexes and prevention of therapeutic resistance are important for future studies (Fig. 3B).

Aside from SWI/SNF remodelers being implicated in DNA damage repair mechanisms, these complexes have been found to localize in the cytoplasm. Due to shuttling of mSWI/SNF subunits between the nucleosome and the cytoplasm, there is interest in identifying the differential localization of these remodelers driving disease phenotypes (Park et al. 2014). Cytoplasmic examples have been noted for SMARCA4 in corticotroph adenomas (Animireddy et al. 2021) and for ARID1B in pancreatic cancer (Bilodeau et al. 2006). Recent work has shown that mSWI/SNF subunits are detectible in the cytoplasm and associate with the ribosomal machinery (Ulicna et al. 2022). SMARCA4 and PBAF subunits were also found to influence translation through their association with the translational machinery. The cytoplasmic SMARCA4 subunit in particular was shown to correlate with disease progression in primary tumors compared with local recurrences and metastatic breast cancer (Ulicna et al. 2022). Together, these results suggest new potential therapeutic targeting for the mSWI/SNF subunits identified in the cytoplasm (Fig. 3B).

SMARCA4 and SMARCA2 are also targets for synthetic lethality. SMARCA4 is required for cancer cell growth through both its bromodomain (reader) and ATPase domain (remodeler) (Rago et al. 2019). The highly selective PROTAC degrader known as ACBI1 was used to bind the bromodomains of SMARCA2/4 for degradation, which effectively reduced cell growth and promoted cell death in cancer cell lines (Farnaby et al. 2019). These specific degraders are of high interest for targeting remodeler complexes that have multiple nonfunctionally redundant subunits that are altered in different diseases. While progress is being made with monotherapies, combination immune therapies with immune checkpoint inhibitors are also being studied for use with mSWI/SNF mutations, particularly with ARID2, polybromo 1 (PBRM1), ARID1A, and SMARC1B in cancer (Fig. 4; Mittal and Roberts 2020). In vitro studies demonstrated that mutations in the PBAF subunit *PBRM1* or loss of BAF180 expression correlated with alterations in JAK–STAT signaling pathways that might contribute to immune checkpoint inhibitor (ICI) responsiveness. Additional studies and trials are needed to demonstrate the breadth of the SWI/SNF mutations’ impact on ICI (Mittal and Roberts 2020).

Additional synthetic vulnerabilities likely exist for other SWI/SNF members. Studies of ncBAF subunits as synthetic-lethal targets for synovial sarcoma and metastatic uveal melanoma are under way, as they share common SMARCB1 cBAF subunit mutations. Depletion of the ncBAF subunit BRD9 inhibits cell proliferation of synovial sarcoma and metastatic uveal melanoma cell lines (Michel et al. 2018). This knowledge led to a clinical trial leveraging a BRD9 degrader in tumors with a loss of SMARCB1 in advanced synovial sarcoma and metastatic uveal melanoma (NCT04965753). Additional clinical trials are in process using SWI/SNF targeted monotherapies and/or in combination therapies in hematologic malignancies such as AML and MDS (NCT04891757 and NCT04879017).

SWI/SNF subunits are also implicated in cardiovascular pathologies. For example, the BAF60c subunit facilitates a physical interaction between *SMARCA4* and the cardiogenic transcription factors TBX4, GATA4, and NKX2–5 (Kadoch et al. 2013). Takeuchi and Bruneau (2009) have shown that increased expression of BAF60c/Smarcd3 plus Gata4 and Tbx5 can direct ectopic differentiation of mouse noncardiac mesoderm into beating cardiomyocytes. SMARCA4 expression is activated in cardiomyocytes in response to hypertrophic stimuli (Hang et al. 2010). Activated SMARCA4 then assembles a BAF/HDAC/PARP chromatin complex on MHC promoters to control the other cardiac transcription factors such as MEF2, SRF, and TEF1 (Molkentin et al. 1998). These findings collectively suggest that BRG1 and SWI/SNF complexes may play a critical role in pathological conditions aside from cancer. Furthermore, a comprehensive evaluation has been conducted comparing the sum of alterations observed in SWI/SNF complexes in neurodegenerative diseases (NDDs). It was shown that nearly 60% of amino acid mutations in SWI/SNF complexes were specific to NDDs and separate from cancer alterations (Valencia et al. 2023). This computational study allows for mechanistic research for NDD alterations for which direct links to disease phenotypes through these alterations have previously been unclear. Aside from cancer and neurodevelopmental disorders, SWI/SNF complexes are altered in HIV, hepatitis B virus (HBV), and inflammatory bowel diseases (IBDs) (Easley et al. 2010; Zhang et al. 2017; Shu et al. 2018; Centore et al. 2020).

The ISWI family ATPases SMARCA1 and SMARCA5 are also implicated across multiple disease types. *SMARCA5* is frequently overexpressed in cancer and contributes to cell proliferation, radiation sensitivity, and DNA damage repair (Fig. 4; Thakur et al. 2022). The DNA instability linked to chromatin remodeling enzymes is associated with various cancer cell types. Depletion of SMARCA5 and other remodeler enzymes MTA2 and INO80 causes R-loop-mediated DNA damage in leukemic cell lines (Bayona-Feliu et al. 2023). INO80 undergoes a unique interaction with nucleosomes by affecting the DNA translocation and H2A–H2B dimer exchange (Fig. 3A; Brahma et al. 2017). The INO80 complex plays an additional role in transcription regulation and can serve as either an activator or repressor depending on the needs of the cell (Conaway and Conaway 2009). As mentioned earlier, DNA damage repair becomes a key driver of disease when impaired, presenting a common theme across various chromatin remodeling complexes. This type of disruption is also seen with the INO80 family subunits, as they are mutated in melanoma, cervical cancer, and NSCLC (Cerami et al. 2012; Vaicekauskaitė et al. 2022).

CHD family remodelers function primarily to organize replication-dependent nucleosome assembly and spacing (Fig. 3A; Alendar and Berns 2021). The nine CHD family members function in cardio development and neurodevelopment, which reveal insights into their disease mechanism contributions (Alendar and Berns 2021). Like the other remodeler complex families, CHD enzymes contribute to disease through DNA damage repair, specifically the nonhomologous end-joining (NHEJ) double-stranded break repair pathway. *CHD7* is frequently mutated in the rare CHARGE syndrome, a genetic disorder characterized by growth retardation; congenital eye, heart, genital, and ear abnormalities; and deafness (Fig. 4). It was shown in multiple epithelial transformed cell lines that p53 binding protein 1 (53BP1) complex is recruited to double-stranded break sites in excess and that histone deacetylases accumulate with CHD7 where 53BP1 is absent to aid in the expansion and recompaction of chromatin (Rother et al. 2020). Additionally, mutations in *CHD7* and *CHD8* and pathogenic variants in BAF subunits have been identified in amyotrophic lateral sclerosis (ALS) and have been linked to several developmental disorders including ASD (Fig. 4; Merner et al. 2016; Zhang et al. 2021a). These particular epigenetic factors that are altered in ASD heavily influence the histone 3 lysine 4 (H3K4) regions of the epigenome that contribute to many developmental and neurodegenerative diseases (Vallianatos and Iwase 2015). Findings from the field on ASD have emerged within the past 10 years with the help of whole-genome sequencing and whole-exosome sequencing studies to reveal even more molecular targets and signaling pathways involved in the epigenetic influence on development that contributes to ASD (Jiang et al. 2022). Recent work highlights that these remodelers are essential for the maintenance of neuronal integrity. In a *Drosophila* model of ALS, CHD1 loss enhances TDP-43 neurodegeneration compared with CHD2, which significantly downregulated TDP-43 in the cortex. These data suggest that the interference of these proteins promotes neurodegeneration and that CHD family members are not interchangeable in their functions when one is altered in disease (Berson et al. 2017). The specificity of these subunits for the chromatin remodelers, with their array of mutations across disease types, allows for unique therapeutic opportunities.

## Conclusion and future perspectives

To properly regulate physiological processes, intricate networks of epigenetic modulators work in concert to control DNA, nucleosome placement/composition, and histone modifications (Millán-Zambrano et al. 2022). The activity of epigenetic modulators (writers, erasers, readers, and remodelers) and their associated modifications extends beyond the presence or absence of PTMs on DNA and histones, as they are also effector proteins. While work is currently under way, the field needs to combine the knowledge across the modifier types and the interface between their associated modifications to fully appreciate how development, cell function, and disease initiation and progression are regulated by their cross-talk. For example, in many situations, these enzymes are redundant, specific, or balanced by another regulator, compounding their complex involvement in homeostasis and disease. Here, we have begun to bridge together classes of enzymes and their mechanism of action in the disease context to emphasize the point that these enzymes and the DNA or histone modification state that they interact with are regularly working together to control biological and pathological processes. For this reason, we need to consider the optimal modalities to target the enzymes and readers. In cases where pathological conditions rely on an enzyme's activity, the direct enzyme inhibition may be the best option. Targeting the enzyme may also be important if the associated complex is critical for normal cell viability. In the case where the loss of function of an epigenetic modulator allows the associated complex(es) to successfully operate and be pathologic, it could be useful to block the associated complex through the use of protein degraders or molecular glues. However, another opportunity to consider targeting allosteric interactions or a critical directly interacting partner is seen with Menin inhibitors. This approach could provide a route to reduce the global toxicity that is seen with epigenetic modulators while allowing more on-target function. This area of investigation will be driven by biochemical, molecular, genetic, and epigenomic methods applied to both normal and disease models.

While not discussed extensively in this review, incorporating knowledge from other fields (namely, metabolism and immunology) will be essential in further understanding these epigenetic mechanisms in physiological and pathological pathways. The activity of epigenetic modulators requires cellular metabolites; thus, understanding the interplay between epigenetics and metabolism in disease contexts is essential. For example, the tumor microenvironment in cancer vastly differs in its metabolic makeup compared with surrounding normal tissue (Qu et al. 2023), which could have a profound impact on the epigenetic modulators as a whole or selectively. Additionally, attention on RNA epigenetics and its role in stress, aging, metabolism, and immunology is a young but exciting field that will open future opportunities for discovery. While not mentioned here, RNA-modifying enzymes have emerging roles in health and disease (Barbieri and Kouzarides 2020), and the cross-talk between these and the modifications discussed in this review will be critical to understand. These studies will help to uncover core epigenetic mechanisms that may reveal further insights into disease pathways (Wu et al. 2024).

For the future of therapeutics, the studies highlighted here have revealed the importance of each class of modifier enzyme and the role they play across multiple disease types. These studies have established that future works should focus on limiting drug toxicity, the method and mode of delivery, and the drug timing or treatment window for vulnerable diseases, as discussed in this review with the examples of doxorubicin and LSD1 inhibitor therapy. While initial attention has been on monotherapies, emerging evidence suggests that combination therapies (e.g., traditional therapies or immunotherapy) with drugs targeting epigenetic regulators will be the most optimal path. Furthermore, leveraging the genetic or metabolic states of the disease and how this impacts the enzyme function will be another key tool to revolutionize the way hard to treat diseases are handled in the clinic. Future studies should also focus on single-cell epigenomic approaches in order to understand the temporal nature of epigenome and genome architecture changes during disease initiation and progression. These advances will be helped with the use of newer technologies such as long-read sequencing methods. Ultimately, while the study of epigenetic modifying enzymes in disease is a relatively young field, it appears highly promising for producing mechanistic insights into disease, unappreciated biomarkers, and novel therapeutics. Continuing on the path to further understanding these modulator enzymes will hopefully create therapeutic options for patients with no current alternative.
